# Whole‐plant microbiome profiling reveals a novel geminivirus associated with soybean stay‐green disease

**DOI:** 10.1111/pbi.13896

**Published:** 2022-08-27

**Authors:** Xiaolin Wang, Mingxing Wang, Like Wang, Huan Feng, Xin He, Shihao Chang, Dapeng Wang, Lei Wang, Jun Yang, Guoyong An, Xiaojie Wang, Lingrang Kong, Zhen Geng, Ertao Wang

**Affiliations:** ^1^ National Key Laboratory of Plant Molecular Genetics, Chinese Academy of Sciences Center for Excellence in Molecular Plant Sciences Institute of Plant Physiology and Ecology, Shanghai Institutes for Biological Sciences, Chinese Academy of Sciences Shanghai China; ^2^ University of Chinese Academy of Sciences Beijing China; ^3^ Northwest A&F University Yangling China; ^4^ State Key Laboratory of Crop Stress Adaptation and Improvement, College of Agriculture Henan University Kaifeng China; ^5^ Zhoukou Academy of Agricultural Sciences Zhoukou China; ^6^ State Key Laboratory of Crop Biology, College of Agronomy Shandong Agricultural University Taian China

**Keywords:** dysbiosis, geminivirus, seed microbiota, soybean, soybean stay‐green syndrome

## Abstract

Microbiota colonize every accessible plant tissue and play fundamental roles in plant growth and health. Soybean stay‐green syndrome (SGS), a condition that causes delayed leaf senescence (stay‐green), flat pods and abnormal seeds of soybean, has become the most serious disease of soybean in China. However, the direct cause of SGS is highly debated, and little is known about how SGS affect soybean microbiome dynamics, particularly the seed microbiome. We studied the bacterial, fungal, and viral communities associated with different soybean tissues with and without SGS using a multi‐omics approach, and investigated the possible pathogenic agents associated with SGS and how SGS affects the assembly and functions of plant‐associated microbiomes. We obtained a comprehensive view of the composition, function, loads, diversity, and dynamics of soybean microbiomes in the rhizosphere, root, stem, leaf, pod, and seed compartments, and discovered that soybean SGS was associated with dramatically increased microbial loads and dysbiosis of the bacterial microbiota in seeds. Furthermore, we identified a novel geminivirus that was strongly associated with soybean SGS, regardless of plant cultivar, sampling location, or harvest year. This whole‐plant microbiome profiling of soybean provides the first demonstration of geminivirus infection associated with microbiota dysbiosis, which might represent a general microbiological symptom of plant diseases.

## Introduction

Plants live in environments rich in microbes, including bacteria, fungi, and viruses. A healthy plant microbiome is crucial for plant survival, nutrient acquisition, abiotic stress tolerance, and disease suppression (Trivedi *et al*., 2020). However, disease can result if a susceptible host plant is in intimate association with a virulent pathogen under favourable environmental conditions (Scholthof, 2007). Interestingly, mutualism and pathogenicity are not inherent microbial properties; instead, these characteristics only exist within certain contexts (Castrillo *et al*., 2017; Chen *et al*., 2020; Fesel and Zuccaro, 2016; Hiruma *et al*., 2016). Seeds represent one of the most crucial stages of a plant's lifecycle and participate in the transmission of microbes from one generation to another and consequently act as the initial inoculum for the plant microbiota (Shade *et al*., 2017). Recent studies showed that seed microbiome have strong, natural priority effects on rhizosphere microbiome and influence the endophytic microbiome of plant aboveground compartments (Moroenyane *et al*., 2021b; Ridout *et al*., 2019). The seed microbiome is thought to be very important for plant health and functioning (Nelson, 2018), and agricultural crop production is severely affected by seed‐borne microbial diseases across the world (Gupta and Kumar, 2020). However, compared to the intensively studied roles of rhizosphere and phyllosphere microbiota in plant nutrition and disease responses (Liu *et al*., 2020; Reinhold‐Hurek *et al*., 2015), our understanding of the fundamental contributions of the seed microbiome to plant health has lagged far behind (Nelson, 2018; Shade *et al*., 2017).

Soybean (*Glycine max* L.) is a major legume crop used for human food, animal feed, and biofuel. Understanding soybean‐associated microbiome might be important to prevent outbreak of soybean diseases and is essential for soybean sustainable agricultural production. Field studies showed that soybean rhizosphere selects a subset of the bulk soil community based on functional cores related to growth promotion and nutrition (Mendes *et al*., 2014), and the bacterial communities in the soybean rhizosphere changed with soil type, soybean genotype, and their growth stage (Liu *et al*., 2019; Moroenyane *et al*., 2021a; Xu *et al*., 2009). Furthermore, the negative interactions between nitrogen‐fixing bacteria and the reduction of *nifH* gene abundance were coupled during soybean development under long‐term nitrogen fertilization (Zhang *et al*., 2021). However, the population size, taxonomical and functional dynamics of a whole‐plant‐associated microbiomes inhabiting the soil and different tissues of the soybean are not fully understood, for example, a comprehensively comparison of bacterial composition and population number between the belowground and aboveground compartments, especially in seeds.

Soybean staygreen syndrome (SGS) (or Zhengqing, the Chinese common name) is a condition in which diseased plants exhibit green stems, flat pods, and aborted seeds at a stage when normal plants have filled pods and are senescing (Figure S1) (Zhang *et al*., 2016). In recent years, SGS has become the greatest challenge for soybean producers in China (Li *et al*., 2019) and is still expanding its geography, including North America (Harbach *et al*., 2016). The direct cause of SGS is highly debated but has rarely been systematically studied. SGS can be induced when pods/seeds are damaged (Zhang *et al*., 2016). Another study from the same group revealed that the feeding of stinkbug (*Riptortus pedestris*) on soybean plants can result in SGS (Li *et al*., 2019). However, we found no direct association between SGS and *R. pedestris* infestation at our sampling sites, where this pest is well controlled by chemicals but SGS continues to occur on a large scale. Therefore, the exact cause of SGS in soybean requires further investigation.

One promising method for identifying the factors causing complex diseases is an integrative approach that combines omics technologies, such as microbiomics, transcriptomics, and metabolomics (Hasin *et al*., 2017). Although this approach has been widely used in studies of human diseases, including cancer (Menyhárt and Győrffy, 2021), cardiovascular disease (Leon‐Mimila *et al*., 2019), and COVID‐19 (Singh *et al*., 2021), it has rarely been used to study plant diseases. Here, we utilized a multi‐omics approach to identify the bacterial, fungal, and viral communities associated with SGS at the main soybean production site in China, using amplicon (16S and ITS), RNA, and small RNA sequencing approaches. We surveyed the composition, function, and dynamics of soybean microbiomes at the bulk soil‐rhizosphere‐root interface and in plant compartments such as stem, leaf, pod, and seed, which have rarely been studied. We focused on elucidating the possible pathogenic agents of SGS in soybean and how SGS affects the assembly and ecological functions of microbiomes that inhabit different soybean tissues. We hypothesized that there is a clear differentiation between different soybean ecological niches, and a pathogenic agent (could be virus, bacterium or fungus) that to be directly associated with the incidence of this disease. Moreover, considering that SGS mainly affects seeds, we hypothesized that the disease had a stronger effect on the microbiome of seeds compared to those of vegetative organs and the rhizosphere.

## Results

### Composition and diversity of bacterial and fungal microbiota associated with diverse soybean compartments

**Figure 1:**
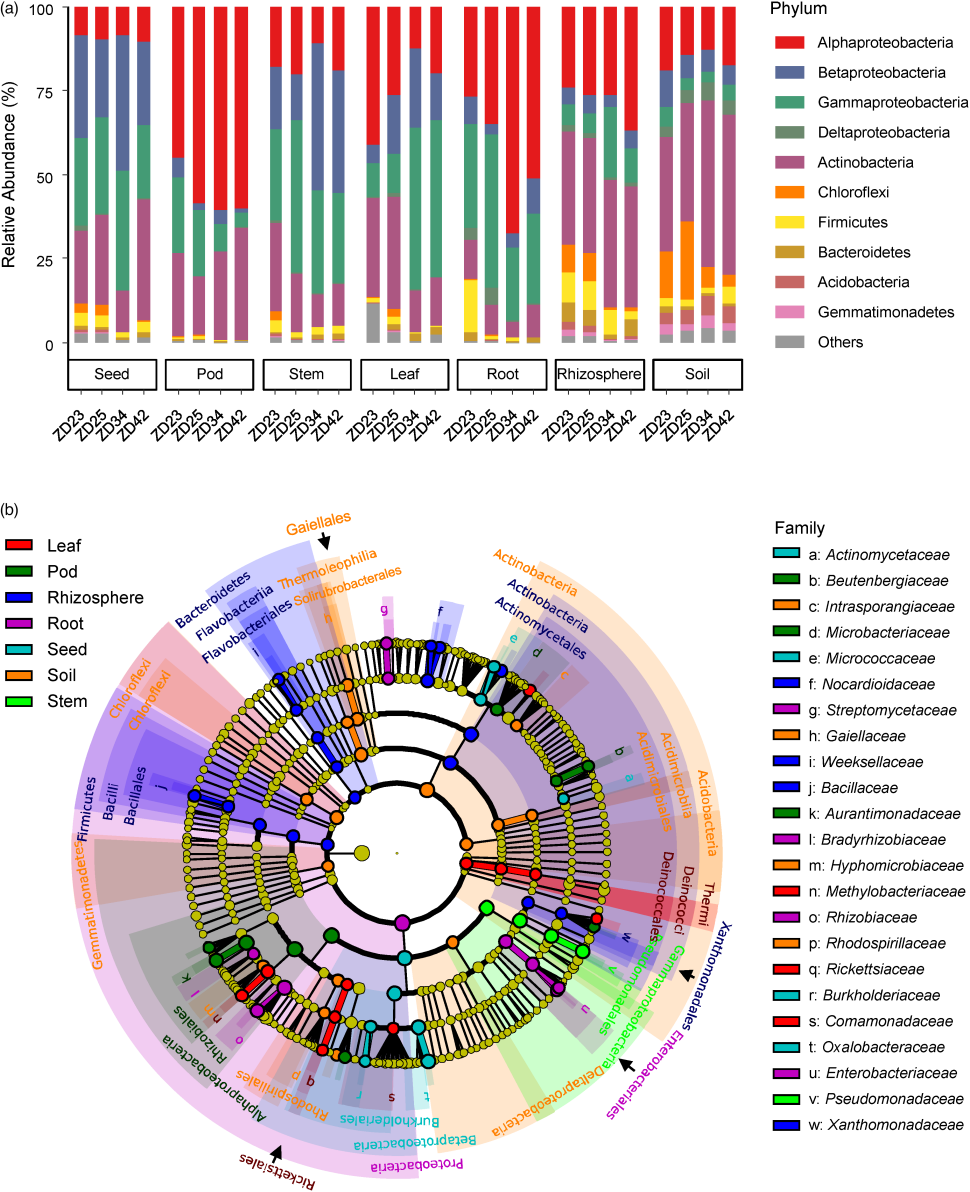
Bacterial composition and biomarkers associated with diverse soybean compartments using 16S *rRNA* gene. (a) Phylum‐level distribution of the bacterial microbiota associated with the seven compartments (seeds, pods, stems, leaves, roots, rhizosphere soils and bulk soils) across four soybean cultivars (ZD23, ZD25, ZD34 and ZD42). Average relative abundance of three biological replicates are displayed in separate stacked bars. (b) LDA effect size taxonomic cladogram comparing all samples categorized by the seven compartments. Significantly discriminant taxon nodes are coloured and branch areas are shaded according to the highest‐ranked variety for that taxon. If the taxon is not significantly differentially represented between sample groups, the corresponding node is coloured yellow. Significantly discriminant taxon from bacterial phylum to order levels are labelled in the cladogram, and that in the family level are labelled in the right.

We first investigated the composition of bacterial and fungal communities living in seven compartments (bulk soil and rhizosphere soil, and roots, stems, leaves, pods, and seeds) of healthy plants (not affected by SGS) of four Zhoudou cultivars (ZD23, ZD25, ZD34, and ZD42) cultivated in the same habitat in Zhoukou, Henan, the main soybean production site in China. At the phylum level, the Alphaproteobacteria and Gammaproteobacteria were gradually enriched along the bulk soil‐rhizosphere‐root gradient (Kruskal Wallis test, false discovery rate‐adjusted [FDR], *P* value <0.05; Figure 1a). Except in pods, Betaproteobacteria were strongly enriched in the endophytic communities of aboveground tissues (Kruskal Wallis test, FDR, *P* value <0.05; Figure 1a). The most highly abundant bacterial families in seeds were bacteria belonging to *Oxalobacteraceae* (17.6% relative abundance) and *Pseudomonadaceae* (16.0%) (Figure S2). The highly diverse fungi that colonized both aboveground and belowground plant tissues mainly belonged to the phyla Ascomycota and Basidiomycota (Figure S3a,b).

We used linear discriminant analysis (LDA) effect size (LEfSe) (Segata *et al*., 2011) to search for statistically significant taxonomic and functional biomarkers for the seven compartments. In total, 79 bacterial biomarkers (LDA >2, *P* < 0.05; Figure 1b and Table S3) were identified. Specifically, *Xanthomonadaceae*, *Nocardioidaceae*, *Bacillaceae*, and *Weeksellaceae* were assigned as biomarkers in the rhizosphere; *Bradyrhizobiaceae*, *Rhizobiaceae*, *Enterobacteriaceae*, and *Streptomycetaceae* in roots; *Comamonadaceae*, *Methylobacteriaceae*, and *Rickettsiaceae* in leaves; *Pseudomonadaceae* in stems; *Aurantimonadaceae*, *Beutenbergiaceae*, and *Microbacteriaceae* in pods; and *Oxalobacteraceae, Micrococcaceae, Actinomycetaceae*, and *Burkholderiaceae* in seeds. The fungal biomarkers for the seven soybean compartments are shown in Figure S3c and Table S4. These taxonomic variability across different compartments were confirmed using Random Forest machine‐learning models (Breiman, 2001) (Figure S4). The functional potential of bacterial communities inferred from PICRUSt2 (Douglas *et al*., 2020) identified 315 MetaCyc pathways with significant differences between compartments (LDA > 2, *P* < 0.05; Figure S5 and Table S5). In particular, pathways involved in *D‐*galacturonate degradation II was significantly more abundant in seed endophytic communities than in the other compartments (Figure S5 and Table S5). Notablely, *D‐*galacturonate is the major component of pectin and an important carbon sources for microorganisms such as *Burkholderiales* that catabolize plant material (Caspi *et al*., 2018), indicating that pectin is an important carbon sources for microorganisms that colonize soybean seeds.

**Figure 2:**
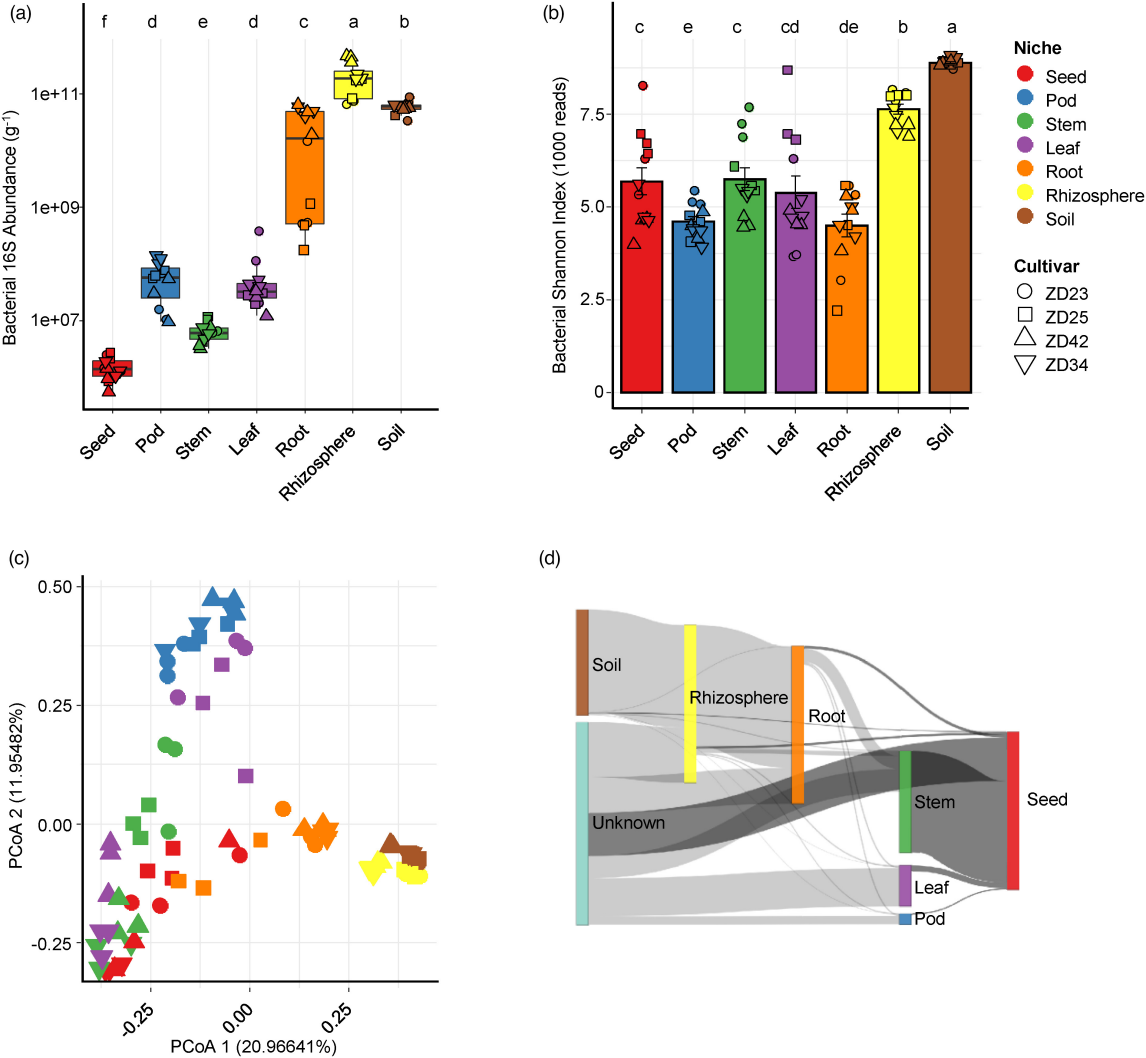
Diversity and dynamics of bacterial microbiota across diverse soybean compartments. (a) Estimated abundance of bacterial 16S *rRNA* genes per gam of sample in diverse soybean compartments. The microbial load was calculated as follows: bacterial 16S rRNA abundance = number of bacterial‐origin reads × (16S synthetic spike copies added/number of spikes‐origin reads). (b) Comparison of bacterial alpha‐diversity between compartments based on the Shannon's diversity index H. (c) Principal coordinate analysis (PCoA) of pairwise Bray–Curtis distances between samples. From (a) to (c), the colour and shape of each point represent the compartment and cultivar, respectively. For (a) and (b), post hoc test is indicated by letters at the top, sample groups with the same letter are indistinguishable at 95% confidence. *n* = 12 biological replicates. (d) Dynamics of the bacterial communities along the soybean compartments as revealed by SourceTracker analysis. Mean proportion of SourceTracker estimates from 12 biological replicates were used for each compartments.

To gain insights into the absolute abundance of bacterial and fungal communities associated with the different soybean compartments, we estimated the microbial loads using spike‐in‐based microbiome profiling methods (Wang *et al*., 2020b). We observed up to a 10 000‐fold variation in bacterial 16S counts (Figure 2a) and a 1000‐fold variation in fungal ITS counts (Figure S6a) between soybean compartments. The highest bacterial and fungal loads were recorded in the rhizosphere (4–6 times higher than those in bulk soil), with an average of 2.12 × 10^11^ and 7.73 × 10^9^ marker gene counts per gram, respectively. The loads of soybean endophytic microbiota gradually decreased from roots to leaves, pods, and stems, with average bacterial 16S counts per gram of 2.49 × 10^10^, 6.64 × 10^7^, 6.27 × 10^7^, and 6.45 × 10^6^ (Figure 2a) and average fungal ITS counts per gram of 1.54 × 10^9^, 5.43 × 10^7^, 4.83 × 10^6^, and 1.79 × 10^6^ (Figure S6a), respectively. Notably, average 1.50 × 10^6^ and 2.90 × 10^5^ counts per gram of bacterial 16S and fungal ITS were estimated in seeds, respectively (Figure 2a and Figure S6a), indicating that seeds were colonized by the fewest microorganisms compared to other plant tissues.

We calculated the alpha and beta diversity metrics to assess the effects of different compartments on soybean microbiota assembly. Significant differences in the alpha diversity of both bacterial and fungal communities were observed among different compartments (Figure 2b, Figures S6 and S7). Interestingly, the alpha diversity values of bacterial and fungal community in aerial endosphere compartments were comparable to or even higher than those in root endosphere (*P* < 0.05, Figure 2b, Figures S6 and S7), suggesting that soybean roots exert stronger selection on microbiomes than aboveground tissues. To assess beta diversity, we performed principal coordinates analysis (PCoA) based on Bray–Curtis distance metrics, which revealed strong clustering of bacterial (PERMANOVA, *R* ^2^ = 0.40, *P* < 0.0001; ANOSIM: *R* = 0.75, *P* < 0.0001) and fungal (PERMANOVA: *R* ^2^ = 0.38, *P* < 0.0001; ANOSIM: *R* = 0.71, *P* < 0.0001) microbiota based on compartment (Figure 2c, Figures S6, S7 and Table S6). Alternative statistics based on weighted UniFrac distances resulted in highly similar conclusions (Table S7). Interestingly, both the bacterial and fungal microbiota of seed samples were intermingled with those of stem samples but positioned far apart from those of pod samples (Figure 2c and Figure S6c), indicating that the seed endosphere microbiota were more likely derived from stems than their neighbouring pod compartment. To test this hypothesis, we calculated the proportion of seed endosphere microbiota derived from other compartments using SourceTracker (Knights *et al*., 2011). The majority of the seed endosphere microbial community was derived from the stem compartment (average 64.54% for bacteria and 44.70% for fungi; Figure 2d, Tables S8 and S9), indicating that horizontal transmission between plant tissues is likely the key driver of seed microbiota assembly.

### SGS results in seed microbiota dysbiosis

**Figure 3:**
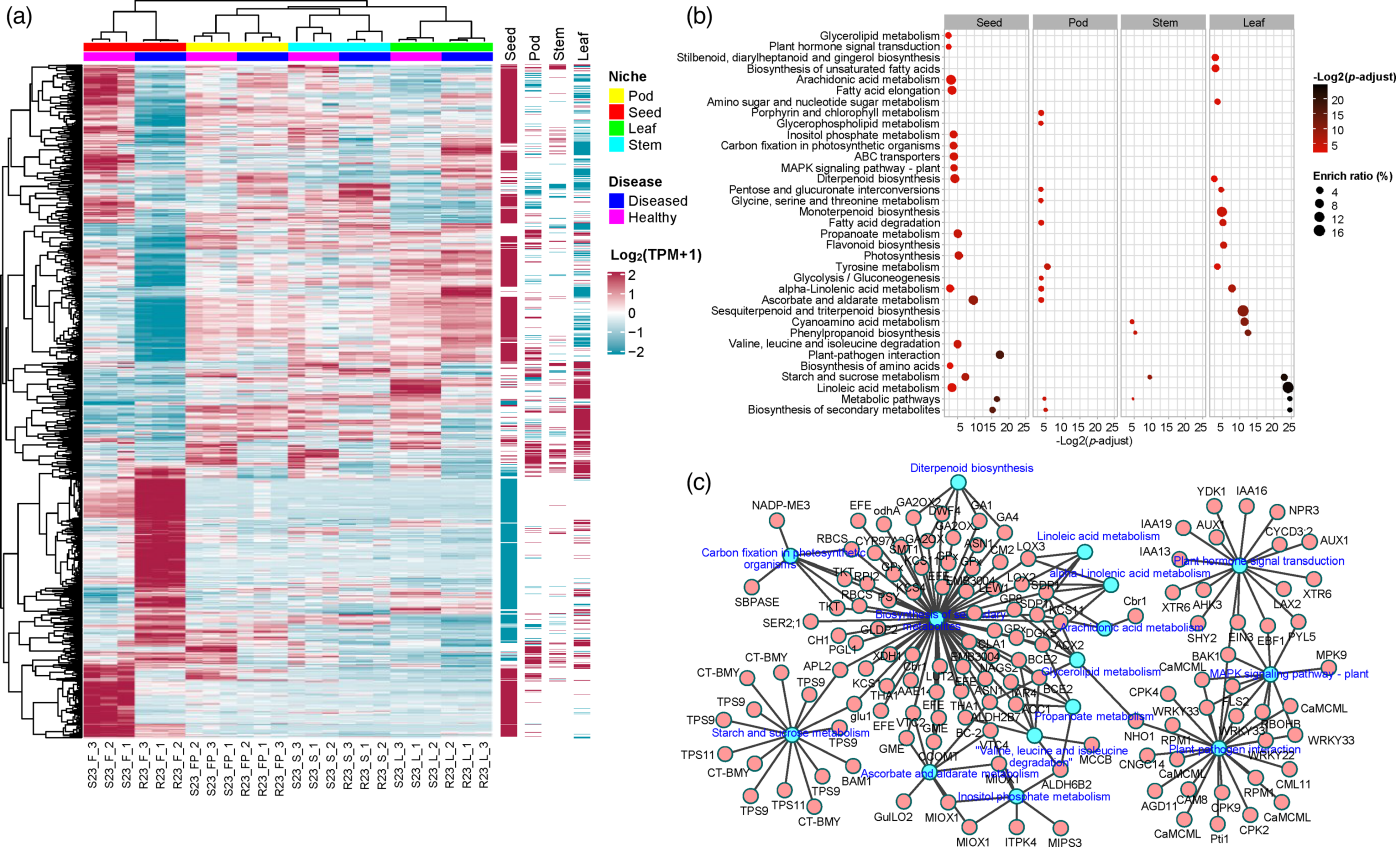
Transcriptome profiles of SGS affected genes and pathways across diverse soybean compartments. (a) Hierarchical clustered heatmap of differentially expressed genes (DEGs) between healthy and SGS diseased samples across diverse soybean compartments. DEGs with ¦log_2_^FC^¦ > 2 (FC, fold change) and adjusted *P*‐value < 0.01 were displayed in the heatmap. Columns on the right indicate DEGs that are categorized as SGS disease induced (red line) and deduced (blue line) gene sets. (b) KEGG pathway enrichment analysis of SGS induced DEGs across diverse soybean compartments. The enriched ratio and FDR‐adjusted enrichment *P*‐value of the pathway were indicated using the size and colour of the bubble points, respectively. (c) The pathway‐gene network of the particular genes induced in SGS diseased seeds in each terms of the enriched KEGG pathways.

To identify the pathogenic agent of SGS in soybean and how SGS affects the transcriptional regulation of soybean, as well as the assembly and ecological functions of soybean‐associated microbiomes, we collected SGS diseased samples which grown in the same habitat and analysed them in parallel with healthy samples. First, we performed RNA sequencing of the aboveground compartments (stems, leaves, pods, and seeds) of both healthy and diseased soybean cultivar ZD23 to explore how SGS affects the transcriptome of soybean. Hierarchical clustering (Figure 3a) revealed that the overall expression profile of SGS‐affected seeds (SGS seeds hereafter) was substantially different from that of healthy seeds. In total, 10 517 differentially expressed genes (DEGs) that responded to SGS were identified in seeds, among which 5854 were upregulated and 4663 were downregulated (Figure S8a and Table S10). KEGG pathway enrichment analysis revealed that upregulated DEGs in SGS seeds were enriched in plant‐pathogen interactions, metabolic pathways, biosynthesis of secondary metabolites, ascorbate and aldarate metabolism, and starch and sucrose metabolism (Figure 3b and Table S11). Downregulated DEGs in SGS seeds were enriched in pathways related to soybean seed oil and protein metabolism, such as linoleic acid, arachidonic acid, glutathione and tyrosine metabolism (Figure S8b and Table S11). To obtain specific information about which genes responded to SGS, we constructed a pathway‐gene network (Figure 3c). Genes related to PAMP‐triggered immunity were highly expressed in SGS seeds, including *CNGCs*, *FLS2*, *BAK1*, *CDPK*, *Rboh*, *Pti1*, *CaMCML*, *WRKY33*, and *NHO1*, indicating that an as yet unidentified pathogen causes the symptoms of SGS. Moreover, genes related to metabolic pathways, biosynthesis of secondary metabolites, ascorbate and aldarate metabolism, and alpha‐linolenic acid metabolism were enriched in SGS pods, genes related to monoterpenoid, sesquiterpenoid and triterpenoid biosynthesis were enriched in SGS leaves (Figure 3b and Table S11). The transcriptional regulation of stem is least affected by SGS, interestingly, we found genes related to circadian rhythm of plant were significantly downregulated in SGS stems (Figure S8b and Table S11), consistent with the green stem disorder of SGS soybean.

**Figure 4:**
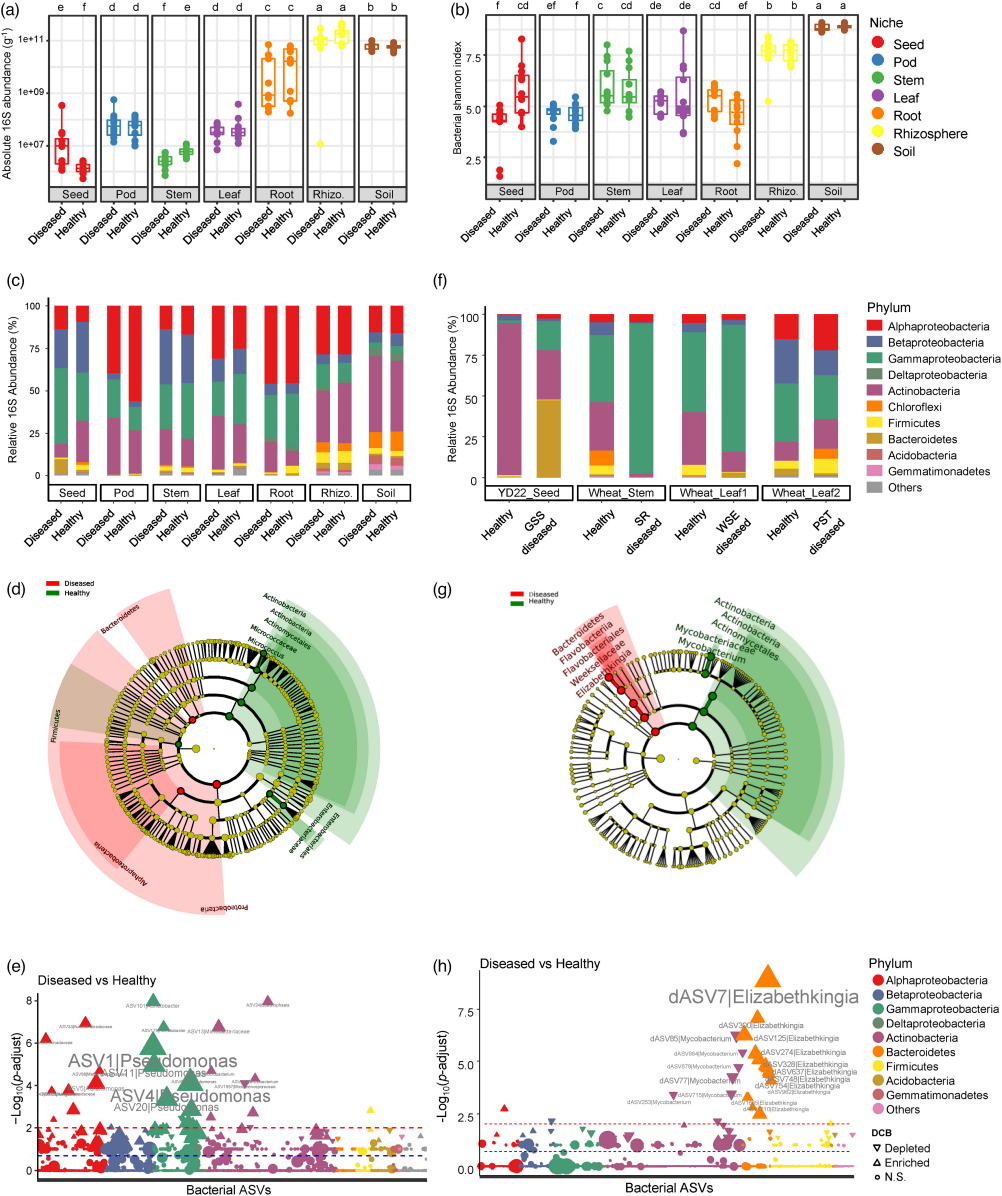
SGS results in seed microbiomes dysbiosis. (a and b) Comparison of the bacterial loads (a) and Shannon's diversity index (b) between healthy and SGS diseased samples in diverse soybean compartments, respectively. Post hoc test is indicated by letters at the top, sample groups with the same letter are indistinguishable at 95% confidence. *n* = 12 biological replicates. (c) Comparison of the phylum‐level distribution of the bacterial microbiota between healthy and SGS diseased samples in diverse soybean compartments. Average relative abundance of 12 biological replicates are displayed in separate stacked bars. (d) LDA effect size taxonomic cladogram comparing bacterial microbiota between healthy and SGS diseased seeds of Zhoudou cultivars. (e) Manhattan plots showing SGS enriched and depleted ASVs in seeds of Zhoudou cultivars. (f) Comparison of the phylum‐level distribution of the bacterial microbiota between healthy and diseased samples associated with diverse plant diseases. YD22_Seed: seeds of soybean cultivar YD22 associated with SGS (*n* = 5 biological replicates); Wheat_Stem: stems of wheat associated with wheat stem rot (SR) complex (*n* = 6); Wheat_Leaf1: leaves of wheat associated with wheat sharp eyespot (WSE) (*n* = 7); Wheat_Leaf2: leaves of wheat associated with wheat stripe rust (WSR) (*n* = 10). (g) LDA effect size taxonomic cladogram comparing bacterial microbiota between healthy and SGS diseased seeds of YD22. (h) Manhattan plots showing SGS enriched and depleted ASVs in seeds of soybean YD22. For (e and h), ASVs that are significantly enriched in SGS diseased seeds are depicted as vertical triangles, that are significantly depleted in SGS diseased seeds are depicted as inverted triangles, otherwise depicted as circles. The red and blue dashed line corresponds to the FDR‐corrected *P*‐value of 0.01 and 0.05, respectively. The colour of each point represents the phylum‐level taxonomic affiliation of the ASVs, and the size corresponds to the baseMean of the ASVs.

We next examined the differences in the diversity and composition of bacterial and fungal communities between healthy and SGS samples. Quantitative microbiome profiling revealed that the loads of bacterial microbiota in SGS seeds increased by 25.66‐fold compared to healthy seeds (diseased, 3.85 × 10^7^; healthy, 1.50 × 10^6^; *P* = 0.0026; Figure 4a). We also notice that bacterial loads in SGS stems decreased by 2.27‐fold compared to healthy stems (diseased, 2.84 × 10^6^; healthy, 6.45 × 10^6^; *P* = 0.0005; Figure 4a). SGS had little or no effect on bacterial loads in other compartments (Figure 4a). The load of fungal microbiota in SGS seeds increased by 3.34‐fold compared to healthy seeds (diseased, 9.68 × 10^5^; healthy, 2.90 × 10^5^; *P* = 0.0021; Figure S9a). Alpha diversity analysis revealed that SGS markedly reduced the diversity of bacterial (Shannon's H: diseased, 4.19; healthy, 5.73; *P* = 0.0047) and fungal (Shannon's H: diseased, 3.12; healthy, 4.16; *P* = 0.00034) microbiomes in the seed endosphere compartment (Figure 4b and Figure S9b). PERMANOVA revealed a small but significant effect of SGS on the structure of the seed bacterial (Bray–Curtis *R* ^2^ = 0.07593, *P* = 0.0431; Weighted Unifrac *R* ^2^ = 0.0647, *P* = 0.1652) and fungal (Bray–Curtis *R* ^2^ = 0.06466, *P* = 0.056; Weighted Unifrac *R* ^2^ = 0.08933, *P* = 0.0118) microbiota (Table S12). A small but significant effect of SGS on the bacterial and fungal microbiota was also observed in pods, but not in other compartments (Table S12), supporting the notion that SGS results in a marked difference in microbial loads and diversity, particularly in soybean seeds.

We then identified bacterial and fungal taxa with different relative abundances between healthy and SGS seeds by performing LEfSe. Notably, Proteobacteria and Bacteroidetes were enriched in SGS seeds, whereas Actinobacteria and Firmicutes were depleted in these seeds (Figure 4c,d), suggesting that the dysbiosis of Gram‐positive (Actinobacteria and Firmicutes) and Gram‐negative (Proteobacteria and Bacteroidetes) bacteria is associated with the incidence of soybean SGS. By contrast, fungal communities were more robust in response to soybean SGS relative to bacterial communities (Figure S9). The microbiota dysbiosis in the seed endosphere compartment was confirmed by BugBase (Gram_negative: healthy, 69.45%; diseased, 84.61%, *P* = 0.0055), whereas other functional pathways and biological phenotypes (such as potentially pathogenic, stress tolerant, and oxygen‐utilizing phenotypes) showed no significant difference between healthy and SGS seeds (*P* > 0.05, Figure S10). Predicted functional profiling using PICRUSt2 revealed that up to 47.6% of MetaCyc pathways were significantly different between healthy and SGS seeds (Table S13, Figures S11 and S12), indicating that SGS induces global changes in both the composition and functions of seed‐associated microbiomes.

We explored the most discriminating bacterial amplicon sequence variants (ASVs) between healthy and SGS seeds that result in microbiota dysbiosis using Manhattan plots (Figure 4e). In the seed endosphere compartment, most SGS‐enriched ASVs belonged to the bacterial classes Alphaproteobacteria and Gammaproteobacteria (Figure 4e). Notably, we determined that the enrichment of *Pseudomonas* ASVs (ASV1, ASV4, ASV9, and so on) was the main cause of seed microbiota dysbiosis (Figure 4e and Table S14). Importantly, although *Pseudomonas* ASVs contributed up to 37% of the total abundance of bacterial microbiota in SGS seeds, its relative abundance in healthy seeds also reached 15%, suggesting that the *Pseudomonas*‐related microbiota dysbiosis in seeds could be a complication rather than the primary cause of SGS.

To test whether the microbiota dysbiosis described above is a robust symptom in the microbiota of diseased seeds, we collected healthy and SGS seeds of a different soybean cultivar (YD22) in a different location (Luoyang, Henan) and sequenced their microbiomes. Surprising, the loads of bacteria increased up to 482.71‐fold in diseased seeds compared to healthy seeds (diseased, 2.52 × 10^10^; healthy, 5.22 × 10^7^; *P* < 0.0001; Figure S13), and microbiota dysbiosis was also observed in SGS seeds (Figure 4f,g). However, the distribution pattern of the bacterial phyla in YD22 was markedly different from that found in the Zhoudou cultivars, with Actinobacteria (93.24% relative abundance) dominating all healthy seeds. This percentage dropped to 30.27% in diseased seeds, which were dominated instead by Bacteroidetes (46.99% relative abundance) (Figure 4f,g). More interestingly, Manhattan plots revealed that the enrichment of *Elizabethkingia* ASVs (dASV7, dASV300, dASV125, and so on) was the main cause of the seed microbiota dysbiosis in YD22 (Figure 4h and Table S15); these ASVs contributed 46.93% and 0.29% of the total abundance of bacterial microbiota in SGS and healthy seeds, respectively.

The dysbiosis was caused by an imbalance of completely different bacterial genera in the SGS seeds of the two soybean cultivars, suggesting that microbiota dysbiosis is not the cause but is instead a common complication of plant diseases. To test this hypothesis, we surveyed microbiome dysbiosis in the tissues of wheat plants with three common wheat diseases: wheat stem rot (SR), wheat stripe rust (WSR), and wheat sharp eyespot (WSE). Although dysbiosis was not observed in WSR‐infected samples, it was observed in the stems of SR‐infected samples and the leaves of WSE‐infected samples, which were both enriched in Proteobacteria and depleted in Actinobacteria (Figure 4f and Figure S14). More strikingly, differential analysis revealed that the enrichment of *Pseudomonas* ASVs was also the main cause of the microbiota dysbiosis in SR‐ and WSE‐infected samples (Figure S15 and Table S15). Together, these results indicate that Gram‐positive and Gram‐negative microbiota dysbiosis is a common microbiological symptom of plant diseases.

### A geminivirus is associated with the incidence of soybean SGS

**Figure 5:**
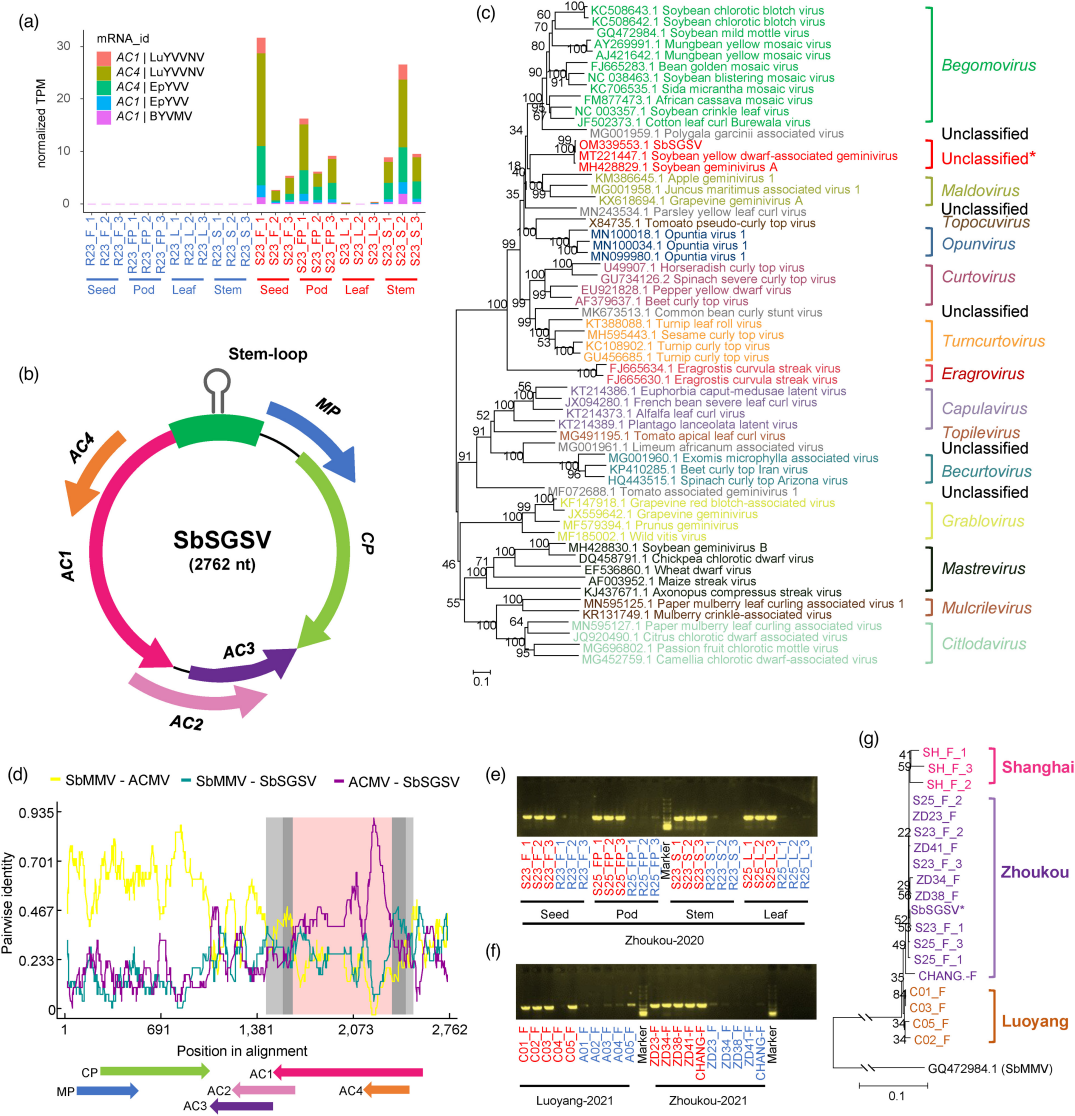
Molecular characterization of a geminivirus associated with the incidence of soybean SGS. (a) *AC1* and *AC4* gene of *Begomovirus* were particularly higher expressed in SGS diseased tissues. Red and blue labels represent diseased and healthy samples, respectively. (b) Schematic representation of SbSGSV genomic organization. Open reading frames organization on the genome sequence are denoted with different colours. (c) Phylogenetic relationships of SbSGSV and representative geminiviruses assigned to the 14 established geminivirus genera based on the nucleotide sequence of the full‐length genome using the neighbour‐joining method. The statistical significance of the branches was determined with a bootstrap of 1000 replicates. (d) Recombination analysis of SbSGSV. The green line indicates the pairwise identity between the SbMMV (Soybean mild mottle virus, GQ472984.1) and SbSGSV, the brown line indicates the pairwise identity between the ACMV (African cassava mosaic virus, FM877473.1) and SbSGSV. The grey region represents the location of predicted breakpoints (99% (grey) and 95% (darkgrey) breakpoint confidence interval, the pink region represents the tract of sequence with a recombinant origin. (e) PCR gel band showing the presence or absence of SbSGSV in the healthy (blue) and SGS diseased (red) tissues. (f) PCR gel band showing the presence or absence of SbSGSV in the healthy (blue) and SGS diseased (red) seeds in YD22 in Luoyang, and in diverse soybean cultivars (ZD23, ZD34, ZD38, ZD41 and a descendant cultivar CHANG18008‐2‐6) in Zhoukou. For (e and f), DNA fragments of 734 bp were amplified using primers Ss_f12‐Ss_r12. (g) Phylogenetic relationships of SbSGSV and amplicon sequences of geminivirus associated with SGS in diverse soybean cultivars across three different locations (Zhoukou, Luoyang and Shanghai). Soybean mild mottle virus (SbMMV, GQ472984.1) was used as outgroup. The neighbour‐joining tree was constructed using the 734 bp sequences amplified by primers Ss_f12‐Ss_r12. The statistical significance of the branches was determined with a bootstrap of 1000 replicates.

Amplicon data analysis revealed that microbiota dysbiosis is associated with the incidence of SGS. However, no bacterial or fungal pathogens were consistently identified in SGS seeds, suggesting that a virus might be the cause of soybean SGS. We therefore surveyed the viral communities associated with SGS. We aligned the RNA‐seq reads to a custom viral genome reference database. A total of 441 genes were mapped to the viral mRNA sequences (Table S16). Among these, 51 genes were differentially expressed between sampling groups, including *Pandoravirus*, bacterial phages, and yellow vein virus (Figure S16). Notably, five genes annotated as *AC1* and *AC4* genes of yellow vein virus were particularly highly expressed in SGS stems, pods, and seeds, but showed almost no expression in healthy samples (Figure 5a and Figure S16).

We subjected the stem, pod, and seed samples to sRNA sequencing for virus identification and performed de novo assembly of the viral genomes. A total of 319 viruses were identified from all samples using VirusDetect (Zheng *et al*., 2017) (Table S17). Differential expression analysis revealed that 63 viral contigs were upregulated in SGS tissues (*P* < 0.05, Figure S17a and Table S17). Notably, almost all upregulated viral contigs were assigned to species in the *Begomovirus* genus (sequence similarity ranges from 86.08% to 95.45%), including yellow vein virus and leaf curl virus (Figure S17a), and all mapped to the same segment (containing a partial *AC1* gene and *AC4* gene) of the reference viral genomes (an example is shown in Figure S17b), suggesting that a novel geminivirus that is not in the reference database is associated with SGS. Therefore, we designed primers based on the longest contig to amplify the full‐length genome of the putative geminivirus. We assembled a full viral genome sequence that we tentatively named soybean staygreen syndrome‐associated geminivirus (SbSGSV, GenBank accession number OM914609; Figure 5b).

The complete genome of SbSGSV consists of 2762 nucleotides (nt), containing two open reading frames (ORFs) in the viral‐sense strand and four ORFs in the complementary‐sense strand (Figure 5b). The size of the genome and the organization of the ORFs are similar to those of members of the *Begomovirus* genus (Figure 5b). A BLASTn search of the complete genome sequence of SbSGSV confirmed its geminiviral origin and its strongest resemblance to soybean yellow dwarf‐associated geminivirus strain HN (MT221447.1, 99.42% identity) and soybean geminivirus A isolate King (MH428829.1, 98.81% identity) (Figure 5c). A Neighbour‐Joining tree inferred from the aligned complete genome sequences of representative isolates from the various geminivirus genera suggests that SbSGSV does not cluster with geminiviruses belonging to any of the 14 established genera (Roumagnac *et al*., 2021) (Figure 5c). Moreover, recombination analysis revealed a possible recombination event in the complete genome of SbSGSV (Figure 5d). This recombination event was located at the 1543–2498 nt region, which included the entire *AC1* ORF, with soybean mild mottle virus (SbMMV) identified as a possible major parent and African cassava mosaic virus (ACMV) as a minor parent (RDP: average *P* value, 1.412 × 10^−14^; Bootscan: average *P* value, 2.340 × 10^−16^; Figure 5d). Hence, the SbSGSV group likely represents a novel viral genus arising from recombination.

To verify whether the identified geminivirus SbSGSV is associated with SGS, we designed primers based on the complete genome of SbSGSV and tested these primers using separate total DNA samples from seeds, pods, stems, and leaves. Notably, amplicons approximately 700 bp long were identified from almost all SGS soybean samples, but no or very weak bands were amplified in the asymptomatic soybean samples (Figure 5e). We investigated the presence or absence of SbSGSV in soybean cultivar YD22, which was collected from a different location (Luoyang, Henan), and in other cultivars (ZD23, ZD34, ZD38, ZD41, and descendant cultivar CHANG18008‐2‐6) from the same location (Zhoukou, Henan), as well as an unknown cultivar that we collected in Shanghai; all samples were collected a year later than our original experiment. PCR analysis of DNA from seeds showed that almost all SGS seeds produced a positive band, whereas no or weak bands were detected in the control (asymptomatic) seeds (Figure 5f). Sequence analysis showed that the sequences of the amplicons were identical among samples; however, the geminivirus had mutated into different genetic variants in different locations (Figure 5g). Taken together, we identified and characterized a geminivirus that is strongly associated with the incidence of soybean SGS in China, regardless of plant genotype, sampling location, or harvest year. We conclude that soybean SGS is likely caused by infection by this geminivirus.

## Discussion

In soybean, microbiome colonization patterns are modulated by compartment niche‐based selection power of the plant (Mendes *et al*., 2014; Moroenyane *et al*., 2021a), plant developmental stages (Moroenyane *et al*., 2021a; Xu *et al*., 2009), soil types, and plant genotypes (Liu *et al*., 2019; Xu *et al*., 2009), mainly based on the characterization of the soybean rhizosphere microbiomes. Here, we conducted a whole‐plant microbiome profiling of the composition, function, and diversity of soybean microbiomes in multiple compartments under agricultural growing conditions. In accordance with the well‐documented plant root and leaf endophytic microbiomes (Bai *et al*., 2015; Bulgarelli *et al*., 2012; Chen *et al*., 2020; Edwards *et al*., 2015; Lundberg *et al*., 2012), the endophytic microbiomes in all soybean compartments examined were dominated by the bacterial phyla Proteobacteria and Actinobacteria and the fungal phylum Ascomycota. However, both the composition and diversity estimates of soybean bacterial and fungal microbiota were highly dependent on plant compartment and were clearly differentiated from each other, consistent with the whole‐plant microbiome studies of poplar (*Populus deltoides*) trees (Beckers *et al*., 2017), tomato (*Solanum lycopersicum*) (Ottesen *et al*., 2013), chilli pepper (*Capsicum annuum*) (Gao *et al*., 2021), and hemp (*Cannabis sativa*) (Wei *et al*., 2021). These differences could be due to host selection, as only adapted taxa can enter and survive in these specialized niches within plant tissues (Hardoim *et al*., 2015).

To our knowledge, our results represent the first clear estimates of bacterial and fungal loads in different tissues of the same plant. Our quantitative microbiome profiling revealed the highest bacterial and fungal loads in the rhizosphere, suggesting that nutrient availability is the major factor that influences rhizosphere microbiota assembly (Qu *et al*., 2020). The population sizes of seed‐associated microbes have rarely been assessed (Nelson, 2018). Our results indicate that while soybean seeds are colonized with the fewest microorganisms compared to the other tissues, they still contain large numbers of microbes with high diversity. In a plant's lifecycle, seed microbiota act as a starting point for the assembly of the plant's microbiota, but our knowledge of seed microbiota assembly remains incomplete (Bever *et al*., 2015; Chesneau *et al*., 2020). A recent study showed that the stem microbiome resembled their parent seed microbiome using soybean seeds grown under both axenic and nonaxenic conditions (Moroenyane *et al*., 2021b). Consistently, here we demonstrated that the endophyte communities of the newly generated soybean seeds are most similar to the bacteria and fungi inhabiting in the stem compartment, suggesting that horizontal transmission between plant tissues is likely the key driver of seed microbiota assembly.

Plant microbiomes are an indicator of plant health (Trivedi *et al*., 2020). The outbreak of SGS has become one of the major challenges to soybean cultivation in China. Compared to soybean green stem syndrome, a condition where the stems of soybean plants remain green when the rest of the plant is senescing, which occurs in the United States and Japan (Hobbs *et al*., 2006), SGS severely damages soybean pods and seeds, causing seed yield losses of up to 80% in China (Li *et al*., 2019). The decreased alpha diversity of bacterial communities is often associated with biotic and abiotic stress, such as pathogen invasion (Shi *et al*., 2019), drought stress (Yang *et al*., 2021), and iron deficiency (Stringlis *et al*., 2018). In this study, we observed significantly lower alpha‐diversity indices in SGS vs. control seeds in the soybean Zhoudou cultivars, whereas no significant difference was detected in soybean cultivar YD22. Further analysis revealed significantly higher microbial loads and Gram‐positive and Gram‐negative bacterial dysbiosis in SGS seeds of all soybean cultivars, as well as in diseased wheat samples with wheat stem rot and wheat sharp eyespot. Moreover, we observed that the dysbiosis was resulted in the abundance shift of different bacterial taxa in different plant diseases, suggested that dysbiosis could be a general microbiological symptom of plant diseases. Based on these results, the microbial loads and dysbiosis in plant microbiomes deserve greater attention in the future to better understand the impact of microbiomes on plant health and ecology.

In mammals, dysbiosis has been associated with the emergence of numerous multifactorial diseases, including inflammatory, autoimmune, metabolic, neoplastic, and neurodegenerative diseases, as has been reviewed in detail (Gomaa, 2020; Levy *et al*., 2017). By contrast, despite the rapidly increasing emphasis on plant microbiomes (e.g., (Bakker *et al*., 2020; Bulgarelli *et al*., 2013; Muller *et al*., 2016; Reinhold‐Hurek *et al*., 2015; Sasse *et al*., 2018; Toju *et al*., 2018)), dysbiosis has only occasionally been reported recently as a critical aspect of the plant microbiome and a potentially important determinant of plant health (Chen *et al*., 2020; Lee *et al*., 2021; Liu *et al*., 2020; Wang *et al*., 2021), and its prevalence in plant diseases was not evaluated in different crops. As to the physiological function of the plant root microbiome parallels the role of the human gut microbiome in host nutrition (Hacquard *et al*., 2015), microbiota dysbiosis perhaps representing a possible paradigm across plant and animal kingdoms. In many cases, it remains to be established whether dysbiosis is a cause or consequence of the disease (Levy *et al*., 2017; Trivedi *et al*., 2020). Interestingly, our previous bacterial community transplantation experiments demonstrated a causal role of a properly assembled leaf bacterial community in phyllosphere health of Arabidopsis (Chen *et al*., 2020). In this study, we found the enrichment of *Pseudomonas* ASVs in Zhoudou cultivars and wheat, and the enrichment of *Elizabethkingia* ASVs in Yundou 22 cultivar, suggesting that dysbiosis is more likely a consequence of the SGS, or an “accomplices” of SGS. However, the role of enriched bacteria in SGS is need to be further explored.

Beside bacterial and fungal diseases, plant viruses can cause devastating diseases and often have wide host ranges (Strange and Scott, 2005). In this study, we identified a geminivirus that was strongly associated with the incidence of soybean SGS, regardless of plant cultivar, sampling location, or harvest year, which we tentatively named SbSGSV. Geminiviruses are among the most devastating plant pathogens worldwide (Roumagnac *et al*., 2021). The documented geminiviruses that attack soybean include mung bean yellow mosaic virus (MYMV) (Malathi and John, 2008), soybean crinkle leaf virus (SCLV) (Iwaki *et al*., 1983), soybean mild mottle virus (SbMMV) and soybean chlorotic blotch virus (SbCBV) (Alabi *et al*., 2010), soybean blistering mosaic virus (SbBMV) (unpublished), and soybean yellow leaf curl virus (SbYLCV) (Du *et al*., 2022); all of these viruses belong to the genus *Begomovirus* of the family Geminiviridae. However, phylogenetic analysis of the full‐length genome showed that SbSGSV is distinct from these documented geminiviruses as well as the 14 established genera of the Geminiviridae family. This indicates that SbSGSV is a recombination‐prone virus, which was further supported by recombination analysis using RDP software. Numerous begomoviruses have been reported to arise from recombination, and the resulting recombinants often have increased fitness or pathogenicity (Ramesh *et al*., 2017). Our results suggesting that the geminivirus SbSGSV is the causal agent of SGS in China, which was verified mutually in a recent study reported that a new geminivirus causes soybean stay‐green disease (SoSGV, which has same length and structure with SbSGSV, and showing >98% complete genome sequence similarity with SbSGSV based on our personal communication with Dr. Xu) (Cheng *et al*., 2022). Further experiments should be performed, such as infectivity assays, net insect experiments, and recombination experiments, to investigate the insect vectors account for the transmission of SbSGSV and to test whether the novel roles of SbSGSV identified in this study were likely acquired as a result of recombination.

## Methods

### Plant materials and samples collection

All samples of soybean Zhoudou cultivars were collected in the main soybean production fields in Zhoukou Farm, Henan Province, China. In August 2020, four soybean cultivars, namely ZD23, ZD25, ZD34 and ZD42, planted in four separated fields, respectively, in the same location (33.57 N, 114.73E) were collected at their late pod‐filling stage. All the four soybean cultivars are new varieties (via classical plant breeding uses deliberate interbreeding of distantly related individuals) with desirable seed yield in Huang‐Huai‐Hai region, where SGS has swept the soybean production (Li *et al*., 2019). At each field, soybean plants that displayed no SGS symptoms (normally pods and seeds) were classified as healthy; plants that showed SGS symptoms (flat pods and aborted seeds) were classified as SGS diseased. Three replicates of healthy and diseased plants were collected at each field. Each replicate consisted of a composite sample obtained by mixing five individual neighbouring soybean plants. While a diseased replicate and a related healthy replicate were collected in the immediate vicinity of the healthy plants. While collecting a plant sample, a bulk soil sample was collected 20 cm away from the root, at a depth of 0–15 cm. The plant samples, and the corresponding bulk soil of each plant, were transported to the laboratory on dry ice. On the same day, seed, pod, stem, leaf, root, and rhizosphere soil samples were collected for each sampling group in the laboratory and stored in triplicate (used for DNA, RNA and sRNA extraction, respectively) at −80 °C until further experiment.

In August 2021, seed samples from both healthy and SGS diseased soybeans of five soybean cultivars, namely ZD23, ZD34, ZD38, ZD41 and a descendant cultivar CHANG18008‐2‐6 planted in the same location were collected at their late pod‐filling stage. Seed samples were stored at −80 °C until further experiment (used for virus detect).

Seed samples both healthy and SGS diseased soybean cultivar YD22 were collected in Luoyang Farm, Hainan Province, China (33.70N, 112.51E) in August 2021. Seed samples were stored in duplicate at −80 °C until further experiment. One is used for DNA extraction and 16S and ITS rRNA high‐throughput sequencing, the other one is used for virus detect.

Moreover, wheat samples associated with three fungal diseases were collected in year 2021. Namely, stem samples associated with wheat stem rot (SR) complex, which caused by *Fusarium* species, and leaf samples associated with wheat sharp eyespot (WSE), which caused by *Geratobasidium cornigeru*, were kindly provided by Lingrang Kong professor in Shandong Agricultural University. Leaf samples associated with wheat stripe rust (WSR), which caused by *Puccinia striiformis*, were kindly provided by Xiaojie Wang professor in Northwest A&F University. Wheat samples were used for DNA extraction and performed as same as soybean leaf and stem samples.

Rhizosphere soil samples were collected as described previously (Wang *et al*., 2020a). To analyse endophytic bacterial and fungal microbiomes associated with soybeans, seeds were first surface sterilized with 5% (v/v) bleach for 1 min, followed with three sonication procedures (30 s at 42 Hz, 30 s pause) in fresh sterile PBS buffer (140 mm NaCl, 2.7 mm KCl, 10 mm Na_2_HPO_4_, 1.8 mm KH_2_PO_4_), which was then blot‐drying to remove surface water and snap‐frozen in liquid N_2_ and stored in −80 °C before DNA extraction. The other plant tissues, namely pods, stems, leaves and roots were performed as same as seeds.

### DNA extraction, amplicons (16S and ITS rRNA) high‐throughput sequencing and processing

DNA extraction was performed using FastDNA SPIN Kit for Soil (MP Biomedicals, Irvine, USA). For quantitative microbiome profiling (QMP), the microbial loads were estimated using the spike‐in based microbiome profiling methods (Wang et al., 2020b). Namely, soil‐ and plant‐associated samples were weighed before DNA extraction, and a certain amount of the synthetic chimeric DNA spikes were added into the DNA samples. For bacterial QMP, 500 pg (about 108 million spike copies) of 16S synthetic spikes were added to each bulk soil and rhizosphere DNA samples, 50 pg were added to each root DNA samples, and 5 pg were added to each DNA samples of aboveground plant tissues (the seed, pod, stem and leaf samples). For fungal QMP, the amounts of ITS synthetic spikes added was one hundredth of that for bacterial QMP. The amounts of spikes added for bacterial and fungal quantitation were different based on the estimated microbial loads of our samples.

For 16S and ITS rRNA high‐throughput sequencing, the V5–V7 regions of bacterial 16S *rRNA* gene sequences were amplified from DNA extracts using the 799F‐1193R (799F: AACMGGATTAGATACCCKG and 1193R: ACGTCATCCCCACCTTCC) universal primers (Bai *et al*., 2015), and the fungal internal transcribed spacer (ITS) region were amplified using the fITS7‐ITS4 (fITS7: GTGARTCATCGAATCTTTG and ITS4: TCCTCCGCTTATTGATATGC) universal primers (Agler *et al*., 2016). The library construction and sequencing (Illumina MiSeq, PE 2 × 300 bp) was conducted by the commercial service provider Shanghai Hanyu Biotech lab (Shanghai, China).

Raw Illumina fastq files were quality filtered and taxonomy analysed using QIIME 2 (Bolyen *et al*., 2019). Briefly, primers of imported sequences were removed via the Cutadapt (Martin, 2011). DADA2 (Callahan *et al*., 2016) was used to filter and denoise sequences, remove chimeras, identify representative sequences of ASVs (amplicon sequence variants) and create an ASV table. For bacterial 16S *rRNA* gene amplicon sequences, representative sequences of ASVs were taxonomically annotated using a pre‐trained Naive Bayes classifier based on the bacterial 16S *rRNA* Greengenes reference database (13_8 release) (DeSantis *et al*., 2006) and the sequences of 16S synthetic spikes. From this taxonomic annotation, all unassigned sequences and sequences annotated as mitochondria, chloroplast and Archaea were removed. Finally, the taxonomic information of the representative bacterial ASVs were annotated in RDP Classifier (using 16 s *rRNA* training set 18) (Wang *et al*., 2007), since it is better updated and provides gene copy number adjustment for 16S *rRNA* gene sequences. For fungal ITS amplicon sequences, representative sequences of ASVs were taxonomically annotated using the fungal ITS classifiers trained on the UNITE reference database (Nilsson *et al*., 2019) and the sequences of ITS synthetic spikes. From this taxonomic annotation, only sequences annotated as Fungi were retained. The metadata and sequencing stats for bacterial and fungal microbiome profiling were provided in Tables S1 and S2. At this stage, QMP of bacteria and fungi were estimated based on the number of spike reads and whole bacterial and fungal reads. Then synthetic spike reads were also removed, and the filtered ASV sequences and the resulting ASV table was then used to determine taxonomic distributions and alpha (Shannon's species diversity and Faith's phylogenetic diversity indexs) and beta (Bray–Curtis distances and weighted UniFrac distances) diversities. For alpha and beta diversity calculations, samples were rarefied to the same number of reads as we described before in Arabidopsis phyllosphere microbiome study (Chen *et al*., 2020). After QIIME2 processing, the filtered representative sequences and ASV tables were then used to determine taxonomic abundances and subsequent statistical analyses in R (see from the “Statistical Analysis and Data Visualization” part in detail).

### RNA extraction, high‐throughput sequencing and processing

To extract RNA for gene expression analysis, 0.5 g of the complete frozen seed, pod, stem, and leaf tissues of plants were pulverized in liquid nitrogen, respectively, and RNAs were extracted using Trizol® Reagent following the manufacturer's protocols (Invitrogen, Carlsbad, CA). The quality and integrity of RNAs were determined by agarose gel electrophoresis and a 2100 Bioanalyzer. The qualified RNAs were treated with DNase (5 U/μL) (TaKaRa, Shiga, Japan) at 37°C for 30 min. The DNase‐treated RNAs were purified by Dynabeads® Oligo (dT)25 (Life Technologies, Carlsbad, USA). The cDNA library construction and sequencing (Illumina HiSeq, PE 2 × 150 bp) was performed by the commercial service provider Shanghai Hanyu Biotech lab at Shanghai (Shanghai, China).

Illumina raw sequencing reads from RNA samples were preprocessed using Trimmomatic (Bolger *et al*., 2014), reads filtered with a quality cutoff of 20 and shorter than 75 bp were discarded. Transcript‐level expression analysis of the clean RNA reads was performed using the Kallisto (Bray *et al*., 2016). Namely, clean reads were mapped to the genome of *Glycine max* Williams 82 (Wm82.a2.v1 https://soybase.org/), and the expression level of each gene mapped to Williams 82 was calculated by Kallisto software.

### Small RNA‐based deep sequencing and processing

Total RNA was extracted from 0.5 g of soybean seed, pod, and stem tissues using Trizol® Reagent following the manufacturer's protocols (Invitrogen, Carlsbad, CA). Small RNAs (sRNAs) were purified from the extracted total RNA using NEBNext Small RNA Library Prep Set for Illumina, which were sequentially ligated with 3′ (5′‐AGATCGGAAGAGCACACGTCTGAACTCCAGTCAC‐3′) and 5′ (5′‐GATCGTCGGACTGTAGAACTCTGAAC‐3′) adapters, reverse transcribed to complementary DNA (cDNA), and PCR amplified. The cDNA library construction and sequencing (Illumina HiSeq, PE 2 × 150 bp) were performed by the commercial service provider Shanghai Hanyu Biotech. The adapter sequences and low‐quality reads were removed using Trimmomatic (Bolger *et al*., 2014) software. High‐quality clean reads were assembled and annotated using the VirusDetect (Zheng *et al*., 2017), a bioinformatics pipeline that can efficiently analyse largescale small RNA (sRNA) datasets for both known and novel virus identification.

Total DNA was extracted from plant seed, pod, and stem tissues using a cetyltrimethyl ammonium bromide (CTAB)‐based extraction procedure, as described in (Murray and Thompson, 1980). Viral genomic DNA was amplified using pairs of adjacent primers. Briefly, primers Ss_f2‐Ss_r2 (Ss_f2: GAGCCTCTGACTTACTGCCG and Ss_r2: CTATCCCCAACCAGGTCAGC), designed based on assembled viral contigs identified by VirusDetect; and then Ss_f11‐Ss_r11 (Ss_f11: TGATCCGCAAAACCCACGTA and Ss_r11: TAATCCCCATAACCCCCGGT), Ss_f12‐Ss_r12 (Ss_f12:GGACTCCGTCAGCAACTGAA and Ss_r12: GTCACCATCACGAACCCCTT), and Ss_f13‐Ss_r13 (Ss_f13: ATTCCCCCGTGCGATAATCC and Ss_r13: AGCTAAATCCAGCTCCGACG) based on the matches of Ss_f2‐Ss_r2 amplicon in NCBI BLASTN searches and the circular DNA molecules of geminivirus. The full‐length genome sequence of virus was finally assembled base on the amplicons of Ss_f11‐Ss_r11, Ss_f12‐Ss_r12 and Ss_f13‐Ss_r13. Open reading frames (ORFs) encoded by the complete viral genome were predicted using ORF Finder (https://www.ncbi.nlm.nih.gov/orffinder/). The reconstructed genome sequences were subjected to the BLASTN algorithm to identify which of the reference virus were most related to.

The representative complete genome sequences of the 14 genera of the family *Geminiviridae* (Roumagnac *et al*., 2021) and the genome sequences of all available documented Geminiviruses attack soybeans were retrieved from the GenBank database and used for Neighbour‐Joining phylogenetic trees construction and recombination analysis. The phylogenetic trees were constructed with 1000 bootstrap replicates using the MEGA11 (Tamura *et al*., 2021). Recombination analysis was carried out using the recombination detection program RDP4 (Martin *et al*., 2015).

### Statistical analysis and data visualization

For microbiota data, PCoA plots based on the Bray–Curtis and weighted Unifrac dissimilarities of bacterial and fungal ASVs were used to visualize niche relationships. Permutational multivariate analysis of variance (PERMANOVA, R‐vegan function adonis) (Anderson, 2001) and analysis of similarities (ANOSIM, R‐vegan function anosim) (CLARKE, 1993) with 9999 permutations were used to test significant differences between groups based on the Bray–Curtis and weighted Unifrac dissimilarities. LEfSe (linear discriminant analysis effect size) (Segata *et al*., 2011) was used to determine the features (differential microbial taxa and function) most likely to explain differences between compartments. PICRUSt 2.0 (Phylogenetic Investigation of Communities by Reconstruction of Unobserved States) (Douglas *et al*., 2020) was used for predicting functional abundances based only on 16S marker gene sequences. DESeq2 R package (Love *et al*., 2014) was used to identify significantly enriched/depleted functional profiles inferred by PICRUSt2 between healthy and SGS diseased samples. SourceTracker 2 (Knights *et al*., 2011) was used to predict the source of microbial communities from other plant compartments to seeds. BugBase (Ward *et al*., 2017) was used to determine high‐level phenotypes (for example, gram positive/negative, biofilm forming, and pathogenic potential) present in microbiome samples.

For RNA‐seq data, Sleuth R package, which makes use of quantification uncertainty estimates obtained via Kallisto (as described in RNA processing) as for accurate differential analysis of genes (Pimentel *et al*., 2017), was used to calculate DEGs between sample groups. Genes with value of ¦log_2_^fold change^¦ > 2 and adjusted *P*‐value <0.01 was defined as the DEGs between sample groups in this study. The hierarchical clustering analyses were performed and visualized with the ‘Heatmap’ function in R from the ComplexHeatmap package (Gu *et al*., 2016), using the sets of DEGs identified in our experiment. DEGs belonging to each compare groups (for example, healthy and SGS diseased seeds) were submitted to KEGG pathway enrichment analyses on the KOBAS platform (Bu *et al*., 2021) to identify over‐represented biological processes. The pathway‐gene network was performed using custom R script and visualized using Gephi (Bastian *et al*., 2009).

For sRNA‐seq data, the DESeq2 R package (Love *et al*., 2014) was used to identify differentially expressed viral contigs between sample groups. Contigs with value of ¦log_2_^fold change^¦ > 2 and *q*‐value < 0.01 was defined as the differentially expressed viral contigs between sample groups in this study. The statistics and visualization were mostly performed using custom R script unless otherwise noted.

## Conflict of interest

The authors declare that they have no competing interests.

## Author contributions

Conceptualization, X.W. and E.W.; methodology, X.W.; investigation, X.W., M.W., L.W., H.F., X.H., S.C., D.W., L.W., J.W., G.A., X.W., L.K., and Z.G.; writing – original draft, X.W.; writing – review & editing, X.W. and E.W.; funding acquisition, E.W. and X.L.; resources, S.C., X.W., L.K., and Z.G.; supervision, E.W.

## Supporting information


**Figure S1** Images depicting the diverse compartment niches and the staygreen syndrome symptoms of soybean.
**Figure S2** The distribution of top 20 bacterial families associated with diverse soybean compartment niches using 16S *rRNA* gene.
**Figure S3** Fungal composition and biomarkers associated with diverse soybean compartment niches using ITS rRNA.
**Figure S4** Random‐forest model detects bacterial and fungal taxa that accurately predict soybean compartment niches.
**Figure S5** Bubble plot of significantly different functional profiles inferred by PICRUSt2 in seven soybean compartment niches.
**Figure S6** Diversity and dynamics of fungal microbiota across diverse soybean compartment niches.
**Figure S7** Phylogenetic diversity of bacterial and fungal microbiota across diverse soybean compartment niches.
**Figure S8** Transcriptome profiles of SGS affected genes and pathways across diverse soybean compartment niches.
**Figure S9** The diversity and composition of fungal communities in response to soybean SGS.
**Figure S10** BugBase analysis compare the proportion of each microbiome with a given phenotype between healthy and SGS diseased samples in diverse soybean compartment niches.
**Figure S11** Bubble plot of significantly enriched functional profiles inferred by PICRUSt2 between healthy and SGS diseased samples in diverse soybean compartment niches.
**Figure S12** Bubble plot of significantly depleted functional profiles inferred by PICRUSt2 between healthy and SGS diseased samples in diverse soybean compartment niches.
**Figure S13** Comparison of the microbial loads (A) and Shannon's diversity index (B) of the bacterial microbiota between healthy and diseased samples associated with diverse plant diseases.
**Figure S14** Microbiomes dysbiosis survey of plant samples associated with wheat diseases.
**Figure S15** Manhattan plots showing disease enriched and depleted ASVs in samples associated with wheat diseases.
**Figure S16** Hierarchical clustered heatmap of differentially expressed viral genes between healthy and SGS diseased samples using RNA sequencing data.
**Figure S17** Viral contigs identified by VirusDetect using sRNA sequencing data.

**Table S1** Bacterial metadata and sequencing stats.
**Table S2** Fungal metadata and sequencing stats.
**Table S3** The bacterial biomarkers categorized by the seven compartment niches are identified by linear discriminant analysis (LDA) effect size analysis.
**Table S4** The fungal biomarkers categorized by the seven compartment niches are identified by linear discriminant analysis (LDA) effect size analysis.
**Table S5** The MetaCyc pathway biomarkers categorized by the seven compartment niches are identified by linear discriminant analysis (LDA) effect size analysis.
**Table S6** PERMANOVA and ANOSIM stats of niche compartment and cultivar effects on bacterial and fungal diversity patterns based on Bray–Curtis distance.
**Table S7** PERMANOVA and ANOSIM stats of niche compartment and cultivar effects on bacterial and fungal diversity patterns based on weighted Unifrac distance.
**Table S8** Estimate the proportion of soybean endosphere bacterial communities derived from other compartments using SourceTracker.
**Table S9** Estimate the proportion of soybean endospherefungal communities derived from other compartments using SourceTracker.
**Table S10** The differentially expressed genes (DEGs) in seeds that responded to SGS.
**Table S11** KEGG pathway enrichment analysis of the differentially expressed genes (DEGs) that responded to SGS across diverse soybean compartment niches.
**Table S12** PERMANOVA and ANOSIM stats of disease effects on bacterial and fungal diversity patterns based on Bray–Curtis and Weighted Unifrac distance, respectively.
**Table S13** The discriminating MetaCyc pathways between healthy and SGS diseased samples across diverse soybean compartment niches.
**Table S14** The discriminating bacterial amplicon sequence variants (ASVs) between healthy and SGS diseased samples across diverse soybean compartment niches.
**Table S15** The discriminating bacterial amplicon sequence variants (ASVs) between healthy and diseased samples across diverse plant diseases.
**Table S16** The genes mapped to the viral mRNA sequences using the RNA‐seq reads.
**Table S17** Viral genomes identified by VirusDetect using sRNA sequencing data.

## Data Availability

Raw microbiota sequencing fastq data reported in this study are publicly deposited in the SRA (Sequence Read Archive) database under the BioProject PRJNA808807 (bacteria 16S rRNA gene accession no. SAMN26106981‐SAMN26107136 and fungal ITS accession no. SAMN26107137‐SAMN26107292). Raw RNA sequencing fastq data reported in this study are available under the BioProject PRJNA808807, accession no. SAMN26566098‐SAMN26566129.
