# Supplementary material for: Whole‐plant microbiome profiling reveals a novel geminivirus associated with soybean stay‐green disease
Source: Plant Biotechnol J. 2022 Aug 27;20(11):2159–73. doi: 10.1111/pbi.13896 (PMC9616524; doi:10.1111/pbi.13896)
Supplement: Supplementary file 1 — Figure S1 Images depicting the diverse compartment niches and the staygreen syndrome symptoms of soybean. Figure S2 The distribution of top 20 bacterial families associated with diverse soybean compartment niches using 16S rRNA gene. Figure S3 Fungal composition and biomarkers associated with diverse soybean compartment niches using ITS rRNA. Figure S4 Random‐forest model detects bacterial and fungal taxa that accurately predict soybean compartment niches. Figure S5 Bubble plot of significantly different functional profiles inferred by PICRUSt2 in seven soybean compartment niches. Figure S6 Diversity and dynamics of fungal microbiota across diverse soybean compartment niches. Figure S7 Phylogenetic diversity of bacterial and fungal microbiota across diverse soybean compartment niches. Figure S8 Transcriptome profiles of SGS affected genes and pathways across diverse soybean compartment niches. Figure S9 The diversity and composition of fungal communities in response to soybean SGS. Figure S10 BugBase analysis compare the proportion of each microbiome with a given phenotype between healthy and SGS diseased samples in diverse soybean compartment niches. Figure S11 Bubble plot of significantly enriched functional profiles inferred by PICRUSt2 between healthy and SGS diseased samples in diverse soybean compartment niches. Figure S12 Bubble plot of significantly depleted functional profiles inferred by PICRUSt2 between healthy and SGS diseased samples in diverse soybean compartment niches. Figure S13 Comparison of the microbial loads (A) and Shannon's diversity index (B) of the bacterial microbiota between healthy and diseased samples associated with diverse plant diseases. Figure S14 Microbiomes dysbiosis survey of plant samples associated with wheat diseases. Figure S15 Manhattan plots showing disease enriched and depleted ASVs in samples associated with wheat diseases. Figure S16 Hierarchical clustered heatmap of differentially expressed viral genes between healthy an [file PBI-20-2159-s001.pdf]

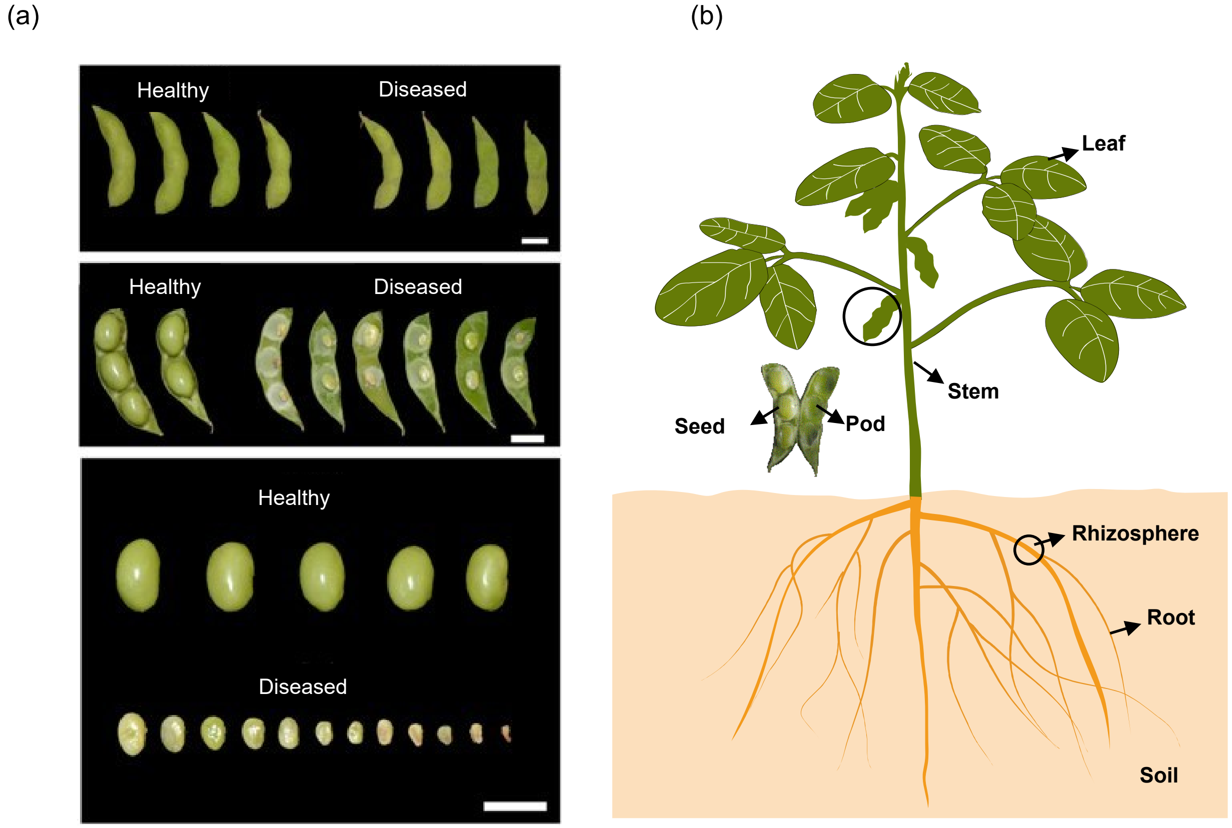

Figure S1 Images depicting the diverse compartment niches and the staygreen syndrome symptoms of soybean. (a) A comparison of normal pods and seeds of healthy soybean to the typical flat pods and aborted seeds associated with soybean staygreen syndrome. (b) Schematic the surveyed plant microbiota members colonizing diverse compartment niches. The surveyed aboveground compartments including the leaf, seed, pod and stem endophytic communities. The surveyed belowground samples are microbial communities associated with bulk soil, rhizosphere soil and root endophytic compartment.

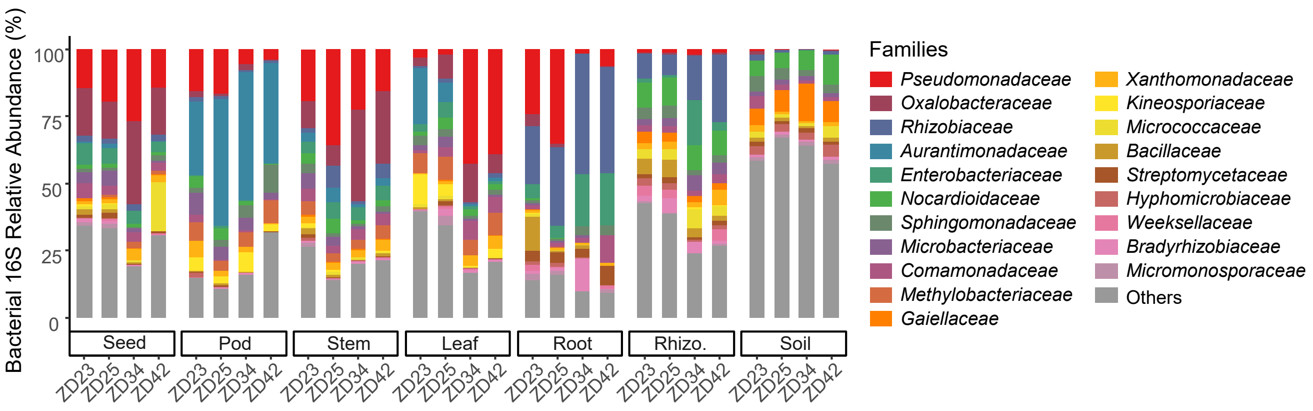

Figure S2 The distribution of top 20 bacterial families associated with diverse soybean compartment niches using 16S *rRNA* gene**.** Average relative abundance of three biological replicates are displayed in separate stacked bars. Major contributing families are displayed in different colours and minor contributing families are grouped and displayed in grey.

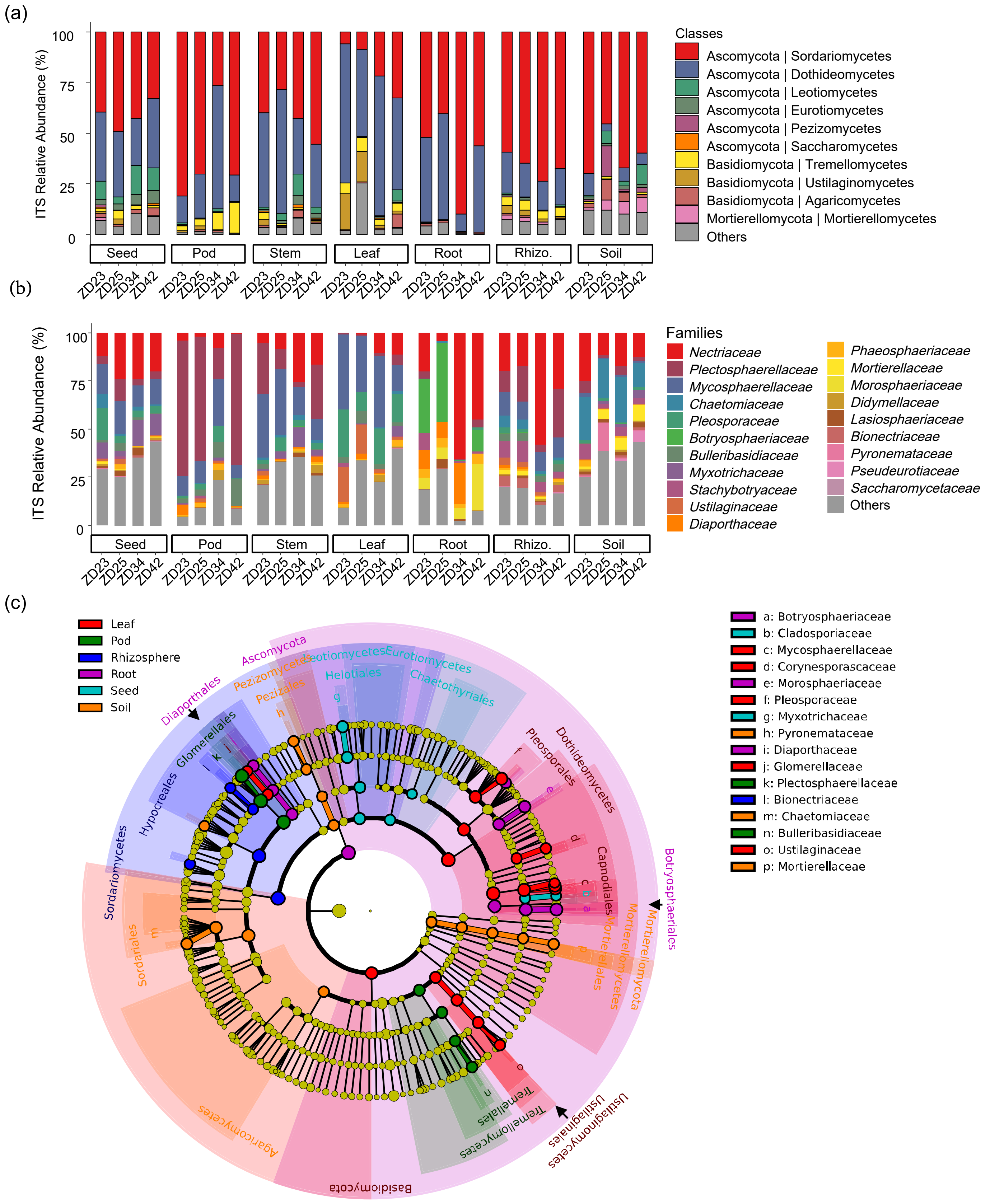

Figure S3 Fungal composition and biomarkers associated with diverse soybean compartment niches using ITS rRNA. Class (a) and family () level distribution of the fungal microbiota associated with the seven compartment niches (seeds, pods, stems, leaves, roots, rhizosphere soils and bulk soils) across four soybean cultivars (ZD23, ZD25, ZD34 and ZD42). Average relative abundance of three biological replicates are displayed in separate stacked bars. Major contributing taxa are displayed in different colours and minor contributing taxa are grouped and displayed in grey. (c) LDA effect size taxonomic cladogram comparing all samples categorized by the seven compartment niches. Significantly discriminant fungal taxon nodes are colored and branch areas are shaded according to the highest-ranked variety for that taxon. If the taxon is not significantly differentially represented between sample groups, the corresponding node is colored yellow. Significantly discriminant taxon from fungal phylum to order levels are labeled in the cladogram, and that in the family level are labeled in the right.

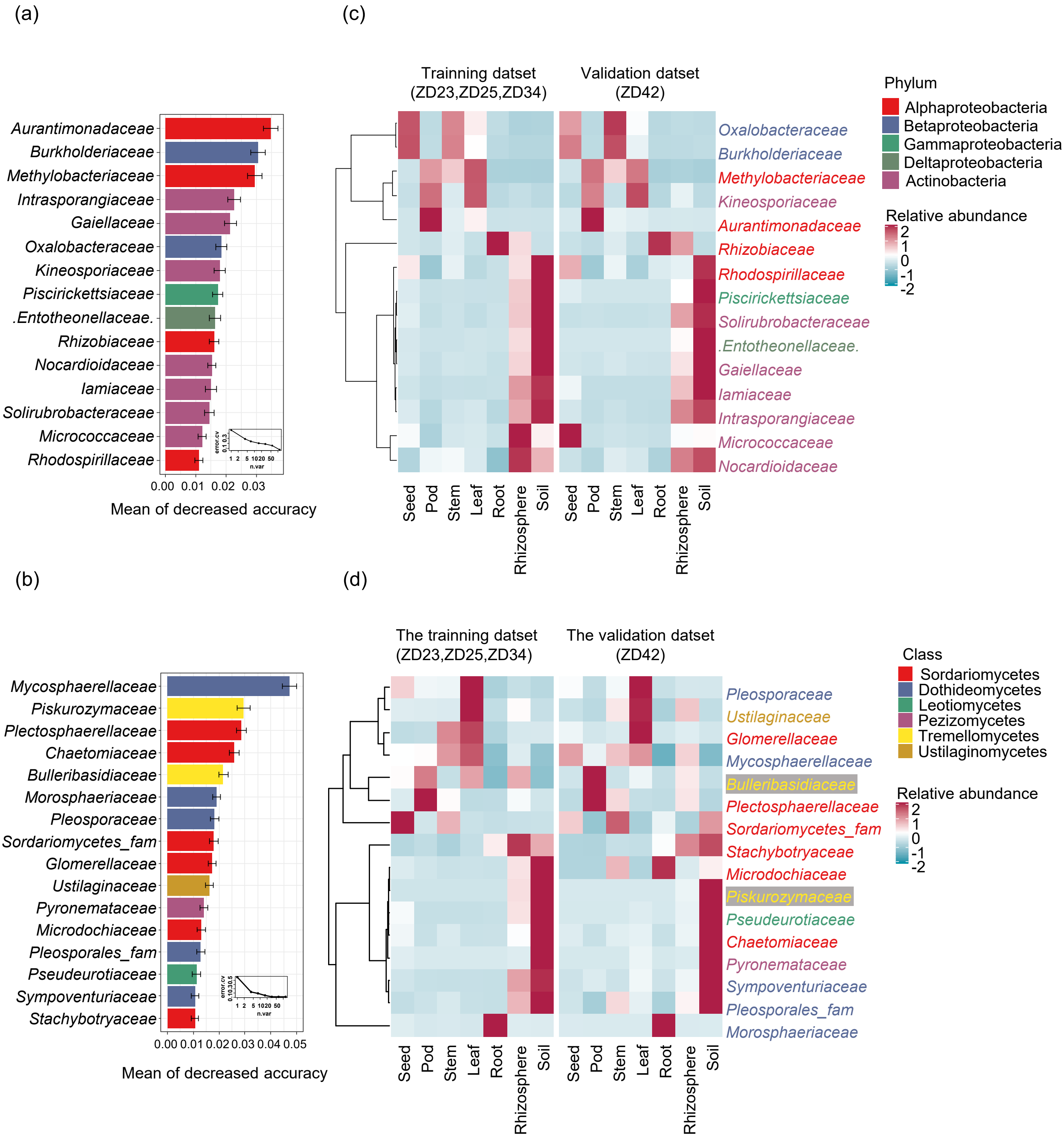

Figure S4 Random-forest model detects bacterial and fungal taxa that accurately predict soybean compartment niches. (a) and (b). The top 15 most relevant bacterial families (**a**) and the top 16 most relevant fungal families () can accurately predicted soybean compartment niches were identified by applying random-forest classification of the relative abundance of the microbiota in soybean cultivars ZD23, ZD25 and ZD34. Biomarker taxa are ranked in descending order of importance to the accuracy of the model. The inset represents 10-fold cross-validation error as a function of the number of input families used to predicted microbiota associated with each soybean compartment niches in order of variable importance. (c) and (d). Heatmap showing the relative abundances of the bacterial (**c**) and fungal (d) biomarker families across diverse soybean compartment niches in the Random-forest trainning datset (soybean cultivars ZD23,ZD25,ZD34), and their relative abundances in the Random-forest validation datset (soybean cultivar ZD42).

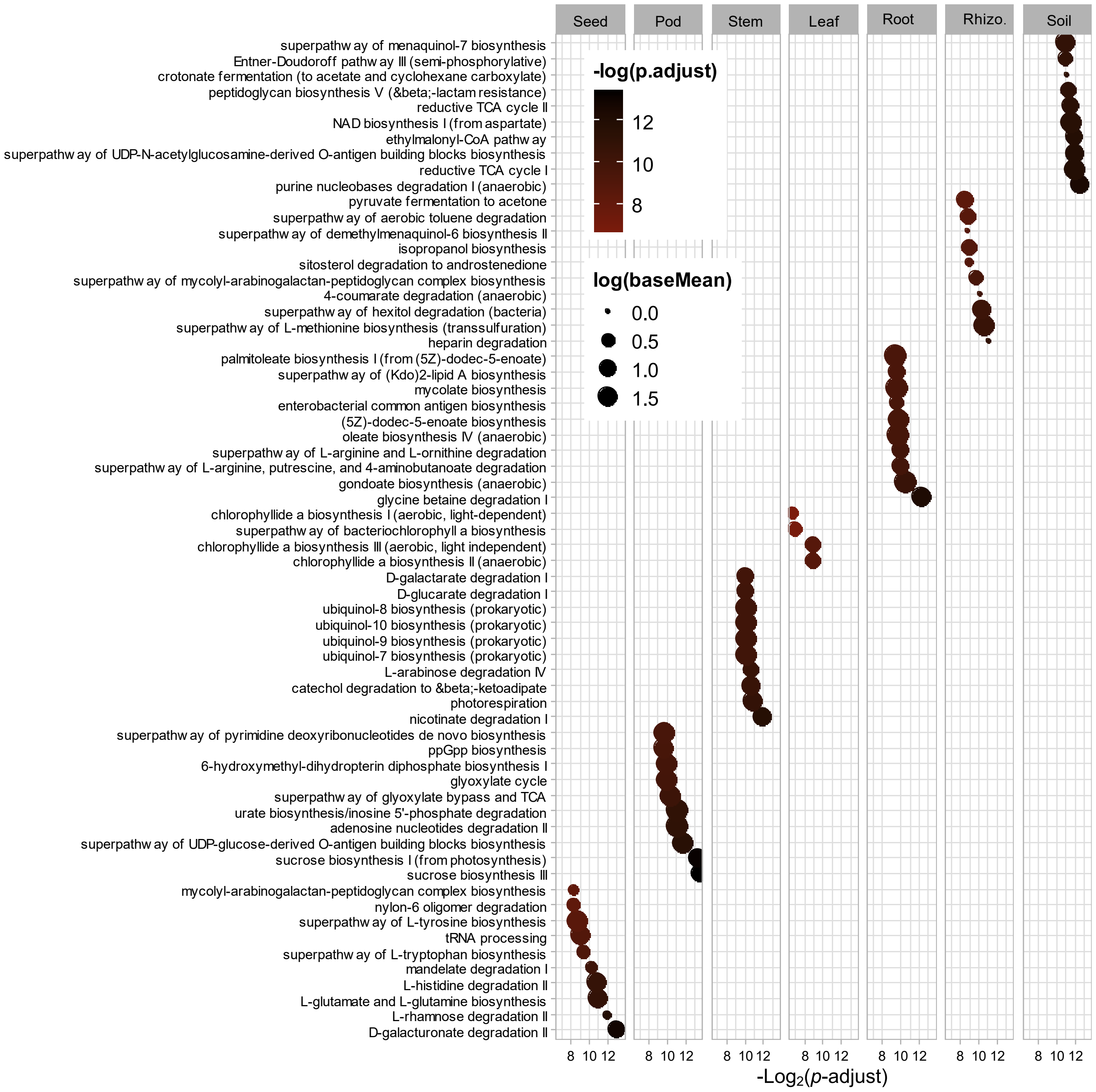

Figure S5 Bubble plot of significantly different functional profiles inferred by PICRUSt2 in seven soybean compartment niches. Each point represents a significantly enriched MetaCyc pathway prediction in a certain compartment relative to all the other compartments identified based on LEfSe analysis. The size of bubble points showing pathway abundances, the color of bubble points showing enrichment significance.

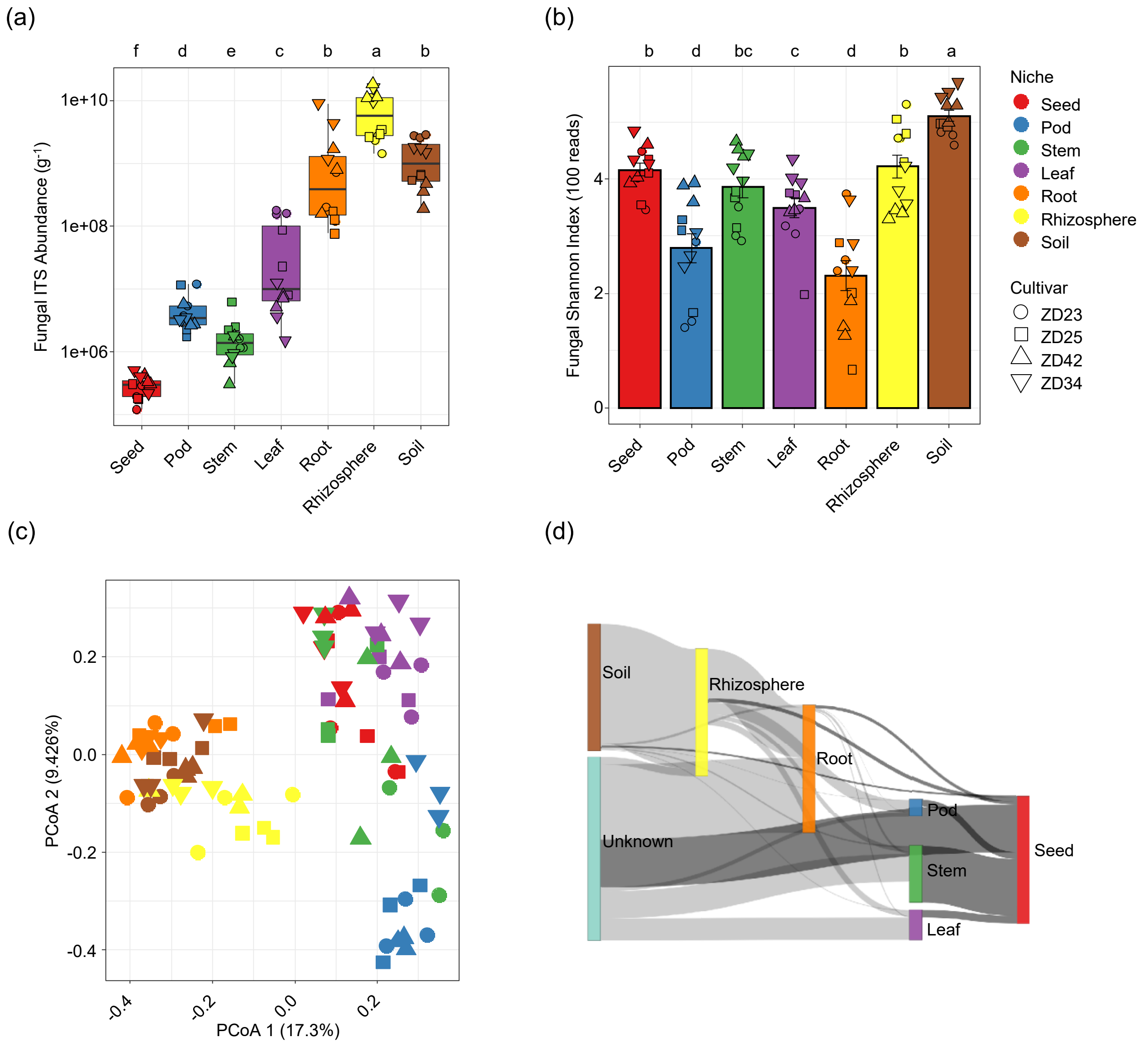

Figure S6. Diversity and dynamics of fungal microbiota across diverse soybean compartment niches. (a) Estimated abundance of fungal ITS rRNA per gam of sample in diverse soybean compartment niches using ITS synthetic spikes. The microbial load was calculated as follows: fungal ITS abundance = number of fungal-origin reads × (ITS synthetic spike copies added/number of spikes-origin reads). (b) Comparison of fungal alpha-diversity between soybean compartment niches based on the Shannon's diversity index H. (c) Principal coordinate analysis (PCoA) of pairwise Bray-Curtis distances between samples. For (a) to (c), the color and shape of each point represent the compartment and cultivar, respectively. For (a) and (b), post hoc test is indicated by letters at the top, sample groups with the same letter are indistinguishable at 95% confidence. n = 12 biological replicates. (d) Dynamics of the fungal communities along the soybean compartment niches as revealed by SourceTracker analysis. Mean proportion of SourceTracker estimates from 12 biological replicates were used for each soybean compartment niches.

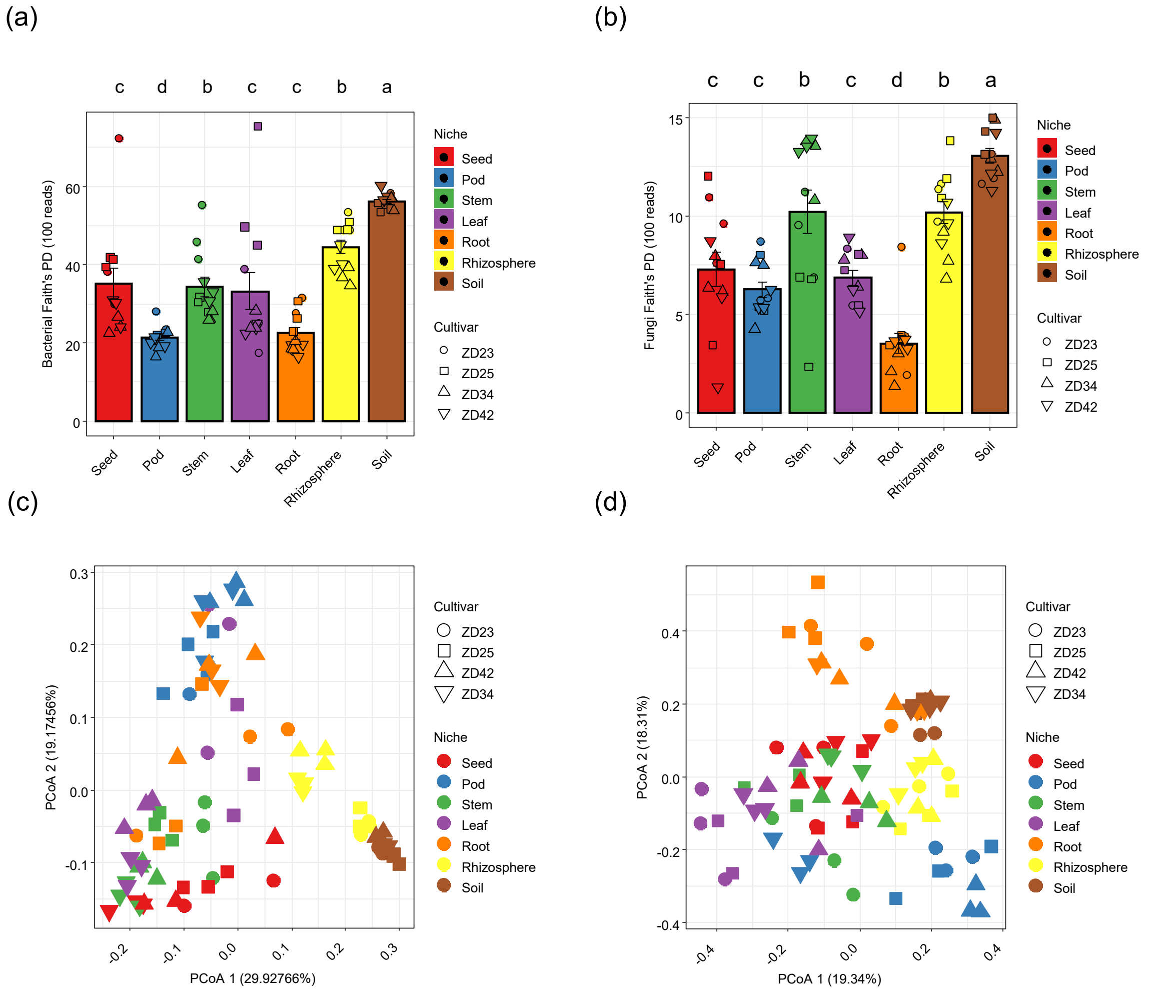

Figure S7 Phylogenetic diversity of bacterial and fungal microbiota across diverse soybean compartment niches. (a) and (b) Comparison of bacterial (a) and fungal (b) alpha-diversity between soybean compartment niches based on the Faith's phylogenetic diversity, respectively. (c) and (d) Principal coordinate analysis (PCoA) of bacterial (c) and fungal (d) pairwise Weighted Unifrac distances between samples, respectively. The color and shape of each point represent the compartment and cultivar, respectively. For (a) and (b)**,** post hoc test is indicated by letters at the top, sample groups with the same letter are indistinguishable at 95% confidence. n = 12 biological replicates.

(a)

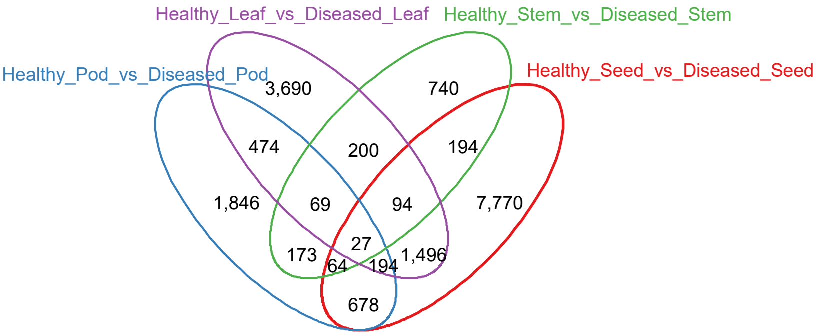

(b)

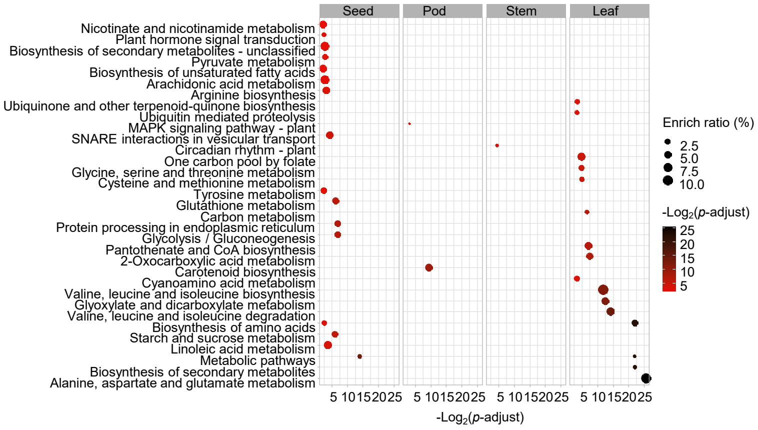

Figure S8 Transcriptome profiles of SGS affected genes and pathways across diverse soybean compartment niches. (a) Venn diagrams of differentially expressed genes (DEGs) between healthy and SGS diseased samples across diverse soybean compartment niches. (b) KEGG pathway enrichment analysis of SGS deduced DEGs across diverse soybean compartment niches. The enriched ratio and FDR-adjusted enrichment P-value of the pathway were indicated using the size and color of the bubble points, respectively.

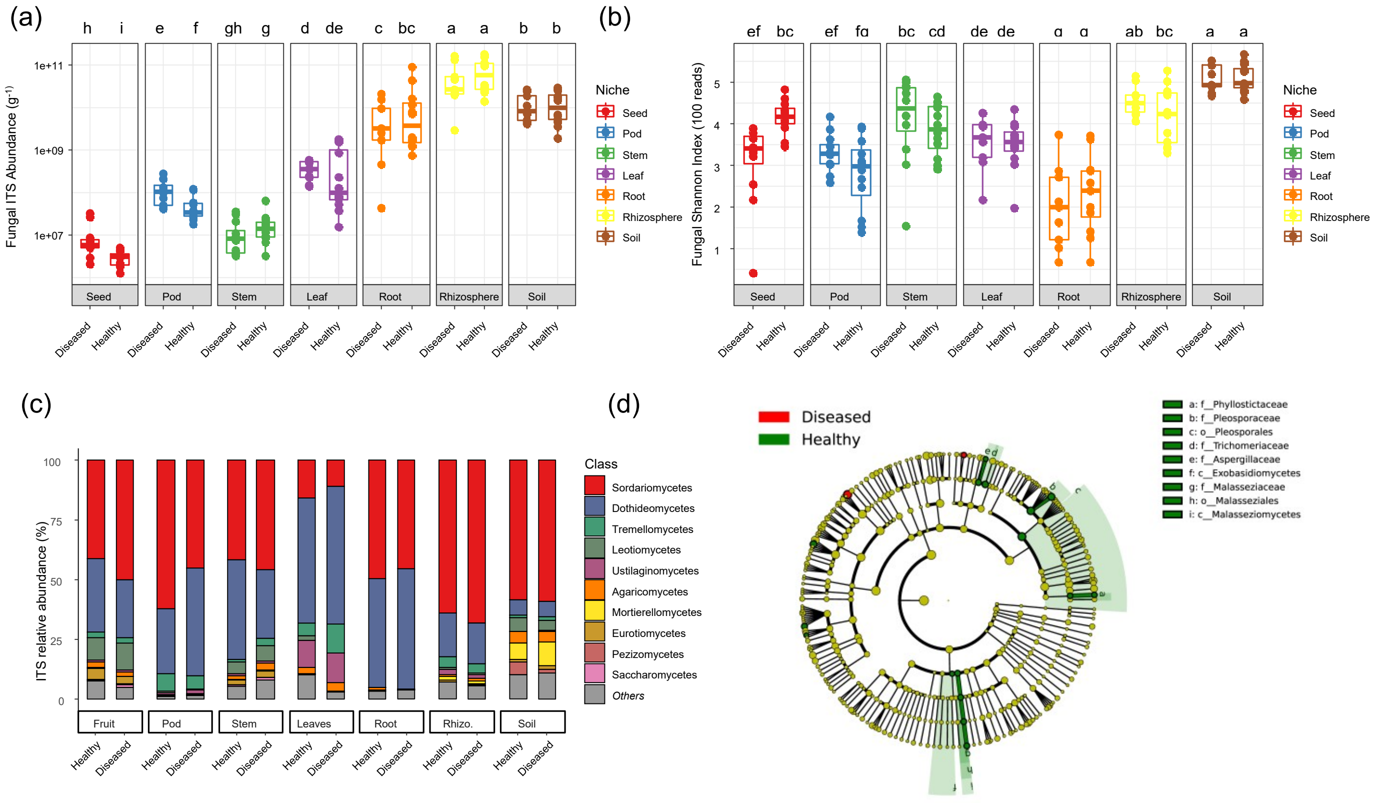

Figure S9 The diversity and composition of fungal communities in response to soybean SGS. (a) and (b) Comparison of the fungal loads (a) and Shannon's diversity index (b) between healthy and SGS diseased samples in diverse soybean compartment niches, respectively. Post hoc test is indicated by letters at the top, sample groups with the same letter are indistinguishable at 95% confidence. n = 12 biological replicates. (c) Comparison of the class-level distribution of the fungal microbiota between healthy and SGS diseased samples in diverse soybean compartment niches. Average relative abundance of 12 biological replicates are displayed in separate stacked bars. (d) LDA effect size taxonomic cladogram comparing fungal microbiota between healthy and SGS diseased seeds of Zhoudou cultivars (ZD23, ZD25, ZD34 and ZD42). Significantly discriminant fungal taxon nodes are colored and branch areas are shaded according to the highest-ranked variety for that taxon. If the taxon is not significantly differentially represented between sample groups, the corresponding node is colored yellow.

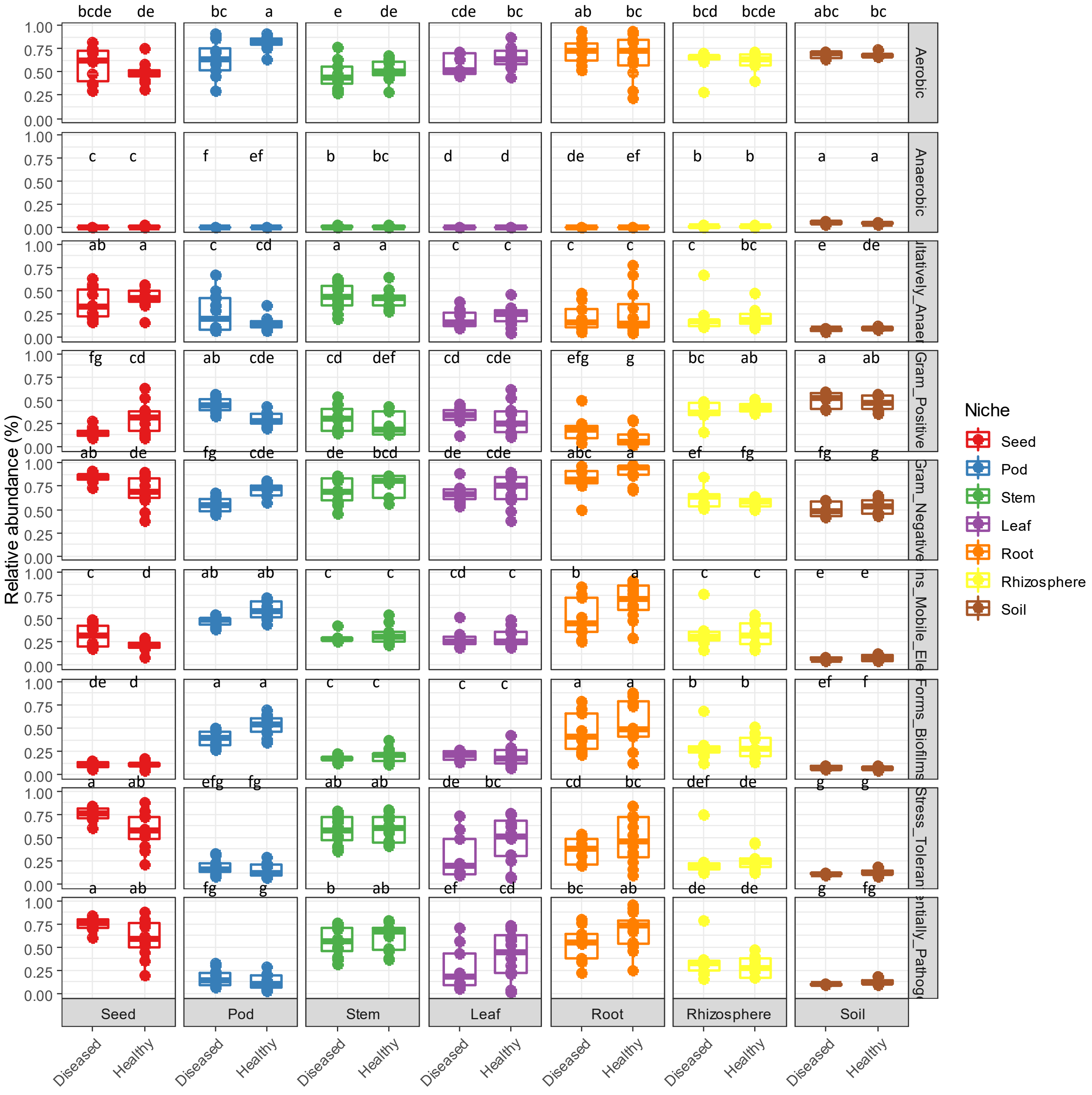

Figure S10 BugBase analysis compare the proportion of each microbiome with a given phenotype between healthy and SGS diseased samples in diverse soybean compartment niches**.** The compared phenotypes from top to bottom of the panel plot are Aerobic, Facultatively Anaerobic, Aerobic, Gram Positive, Gram Negative, Mobile Element Containing, Biofilm Forming, Oxidative Stress Tolerant and Pathogenic Potential, respectively. Post hoc test is indicated by letters at the top, sample groups with the same letter are indistinguishable at 95%confidence. n = 12 biological replicates.

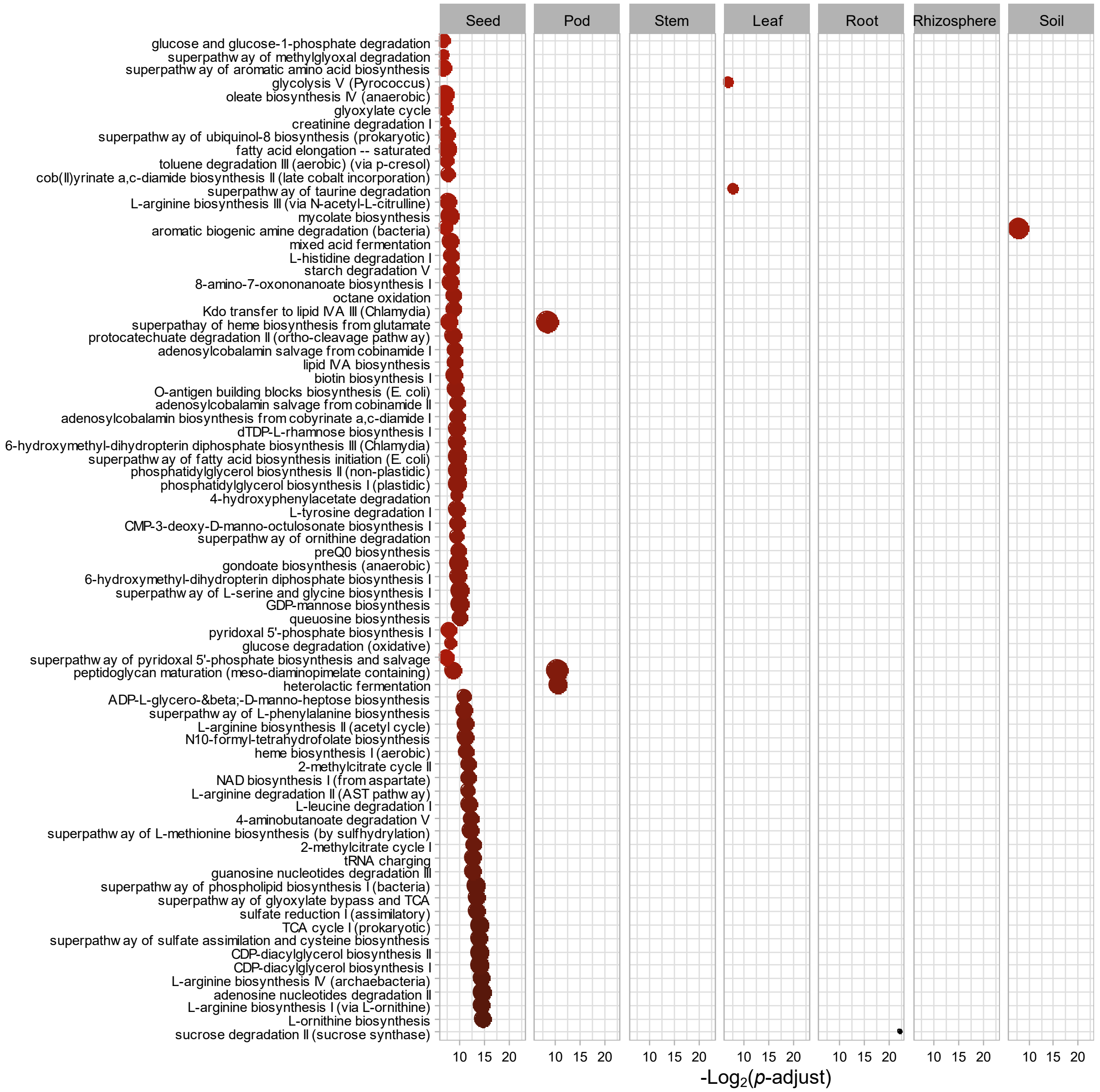

Figure S11 Bubble plot of significantly enriched functional profiles inferred by PICRUSt2 between healthy and SGS diseased samples in diverse soybean compartment niches. Each point represents a significantly enriched MetaCyc pathway prediction in SGS diseased seeds relative to healthy seeds identified based on DESeq2 analysis. The size of bubble points showing pathway abundances, the color of bubble points showing enrichment significance.

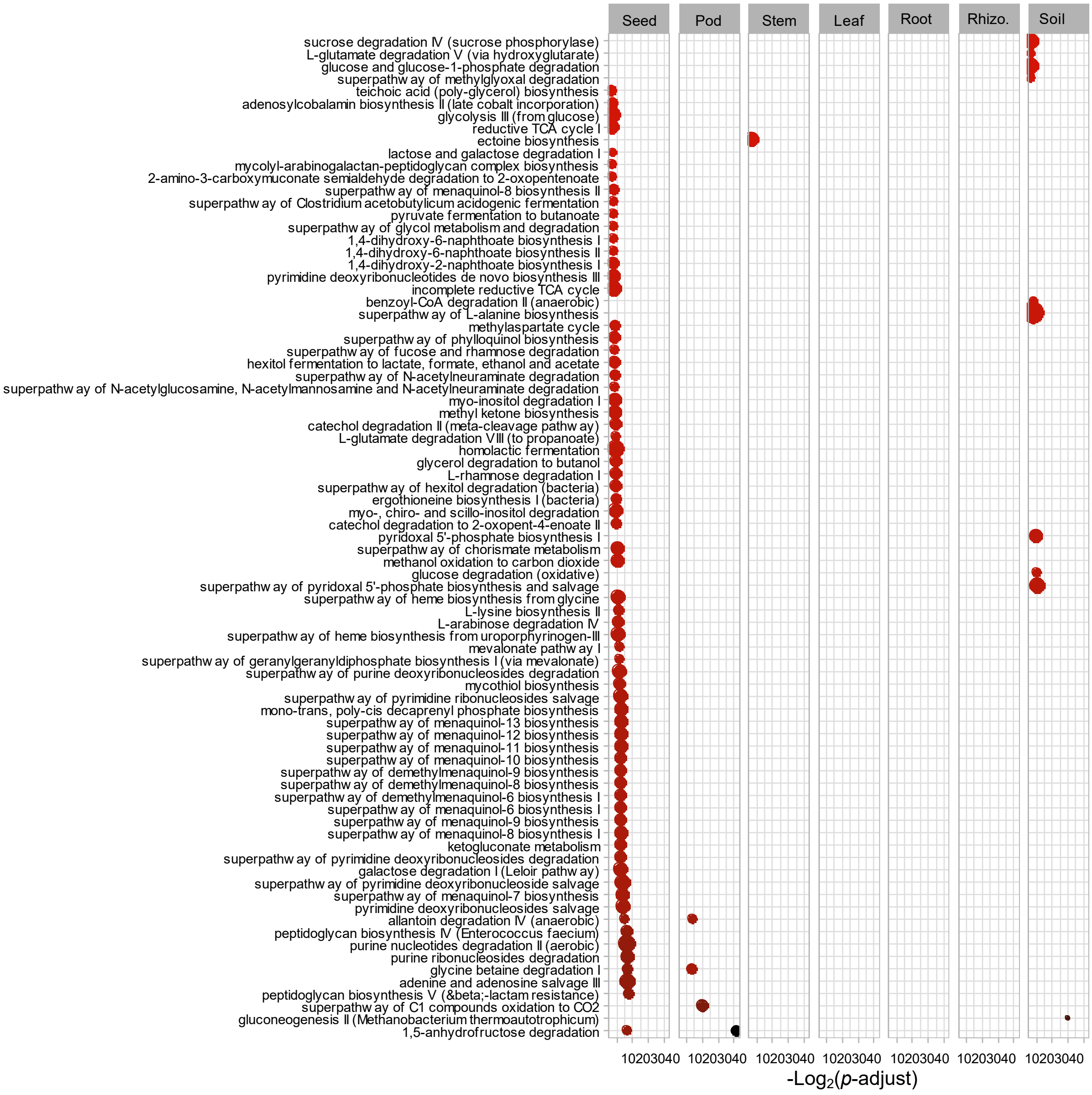

Figure 12 Bubble plot of significantly depleted functional profiles inferred by PICRUSt2 between healthy and SGS diseased samples in diverse soybean compartment niches. Each point represents a significantly depleted MetaCyc pathway prediction in SGS diseased seeds relative to healthy seeds identified based on DESeq2 analysis. The size of bubble points showing pathway abundances, the color of bubble points showing enrichment significance.

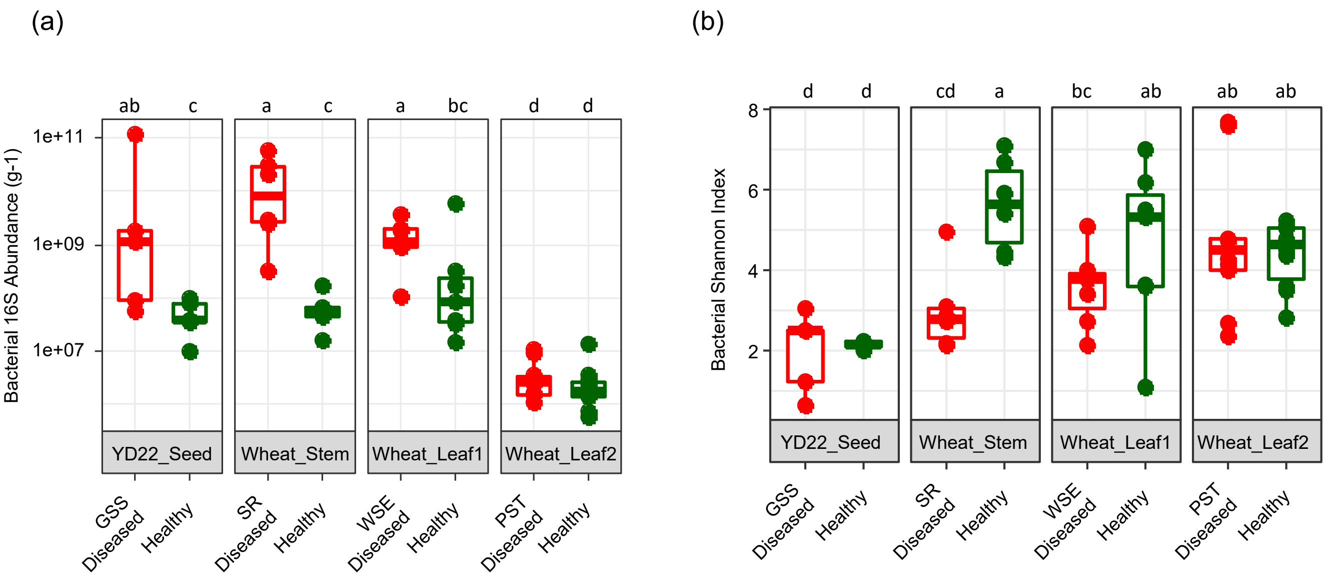

Figure S13 Comparison of the microbial loads (a) and Shannon's diversity index (b) of the bacterial microbiota between healthy and diseased samples associated with diverse plant diseases. YD22_Seed: In seeds of soybean cultivar YD22 associated with SGS (n = 5 biological replicates); Wheat_Stem: in stems of wheat associated with wheat stem rot (SR) complex (n=6); Wheat_Leaf1: in leaves of wheat associated with wheat sharp eyespot (WSE) (n=7); Wheat_Leaf2: in leaves of wheat associated with wheat stripe rust (WSR) (n=10). Post hoc test is indicated by letters at the top, sample groups with the same letter are indistinguishable at 95% confidence.

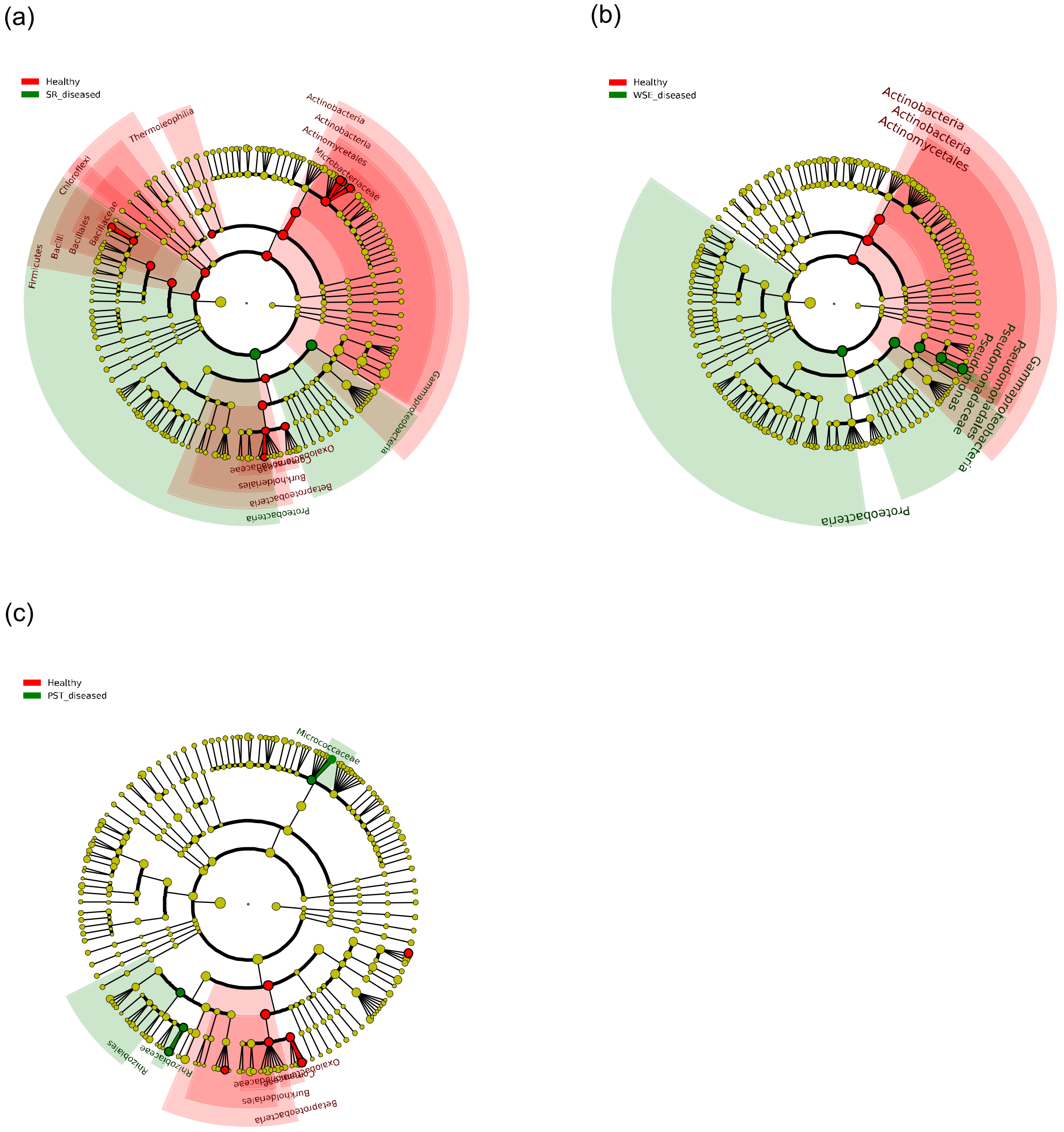

Figure S14 Microbiomes dysbiosis survey of plant samples associated with wheat diseases. **(**a-c**).** LDA effect size taxonomic cladogram comparing bacterial microbiota between healthy and SGS diseased samples associated with wheat stem rot (SR) complex (a), wheat sharp eyespot (WSE) (b) and wheat stripe rust (WSR) (c). Significantly discriminant bacterial taxon nodes are colored and branch areas are shaded according to the highest-ranked variety for that taxon. If the taxon is not significantly differentially represented between sample groups, the corresponding node is colored yellow.

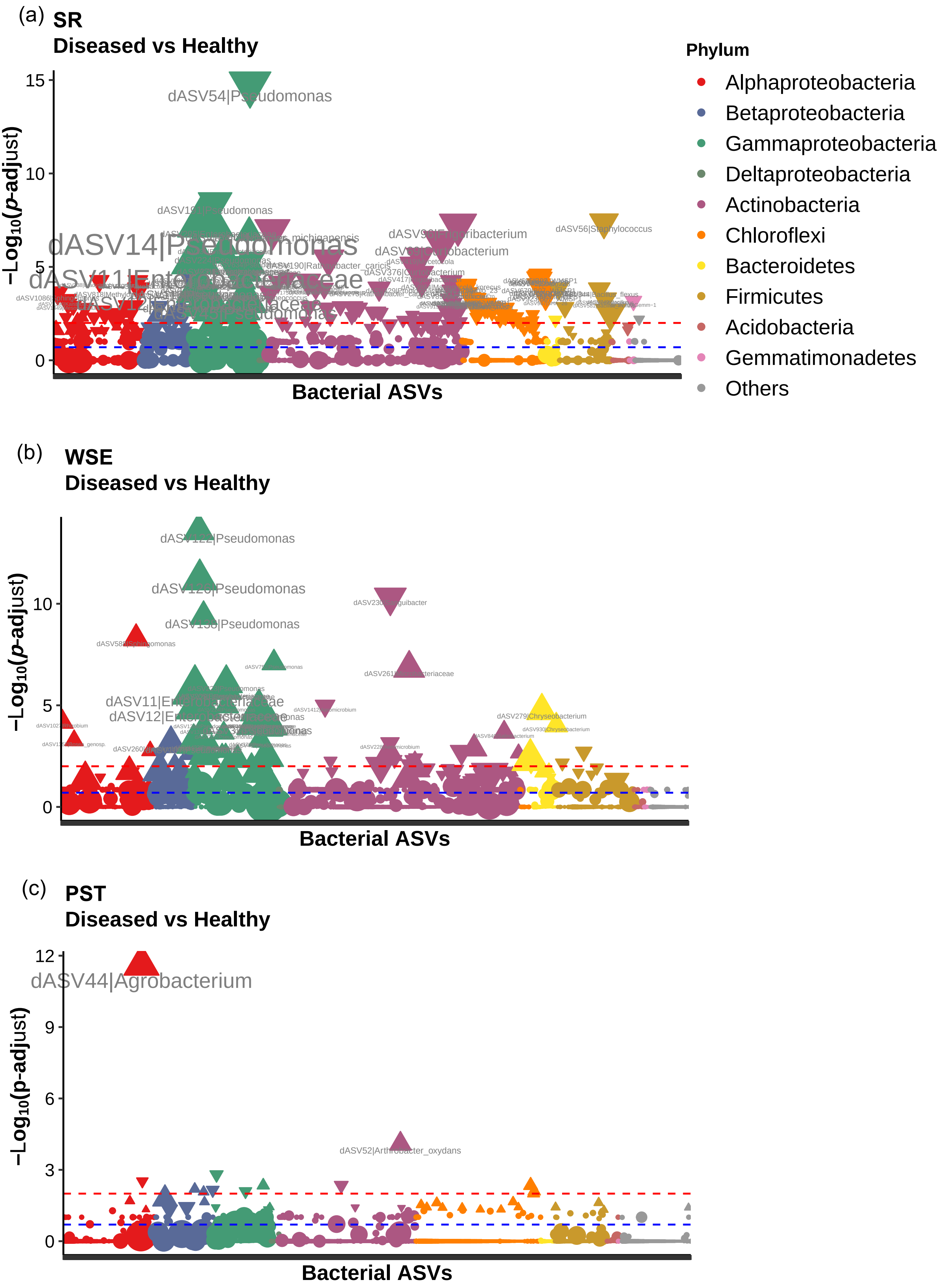

Figure S15 Manhattan plots showing disease enriched and depleted ASVs in sampes associated with wheat stem rot (SR) complex (a), wheat sharp eyespot (WSE) (b) and wheat stripe rust (WSR) (c).

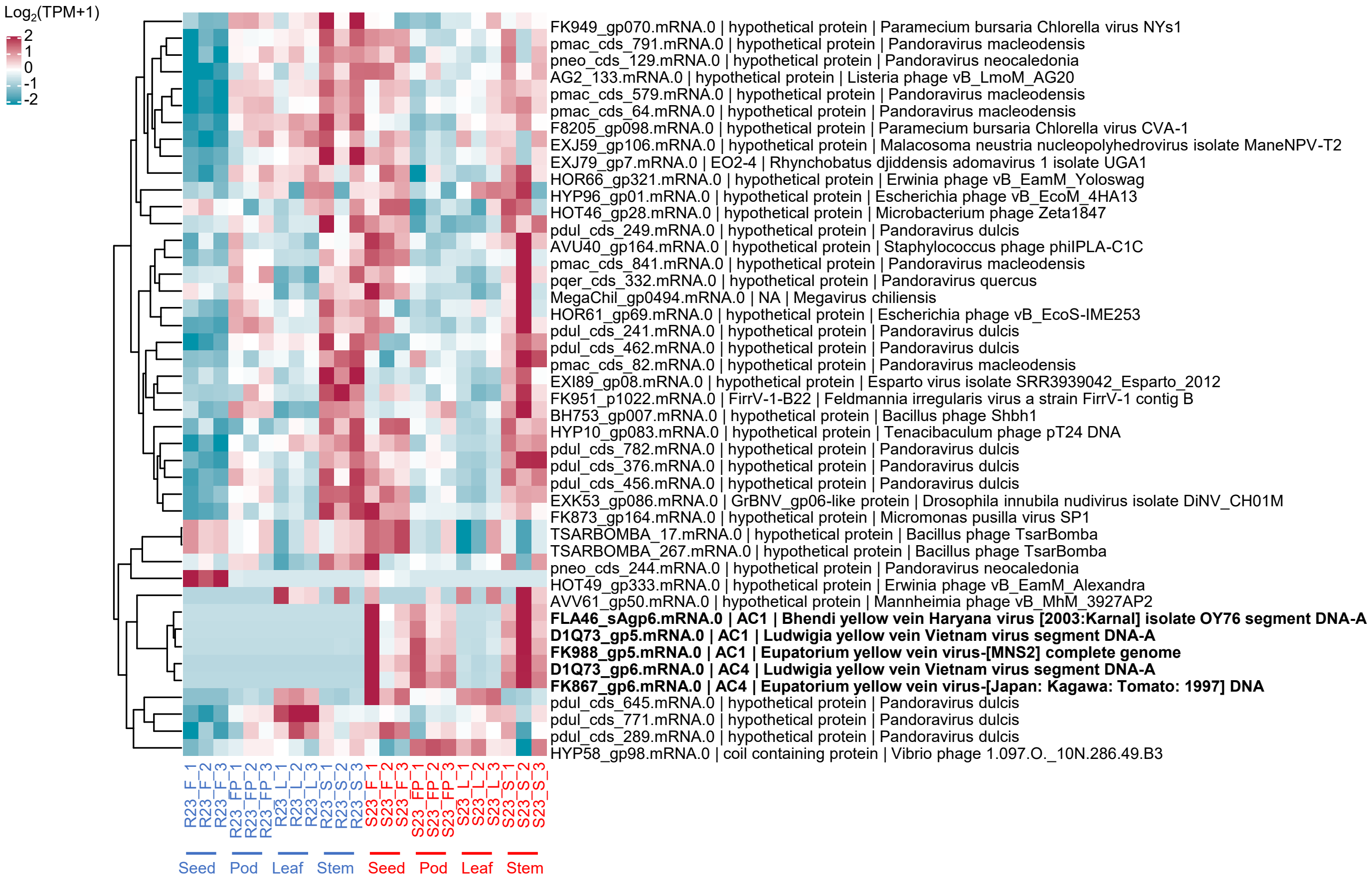

Figure S16 Hierarchical clustered heatmap of differentially expressed viral genes between healthy and SGS diseased samples using RNA sequencing data. Differentially expressed viral genes with |log2FC| > 2 (FC, fold change) and adjusted P-value < 0.01 were displayed in the heatmap. Red and blue labels represent diseased and healthy samples, respectively.

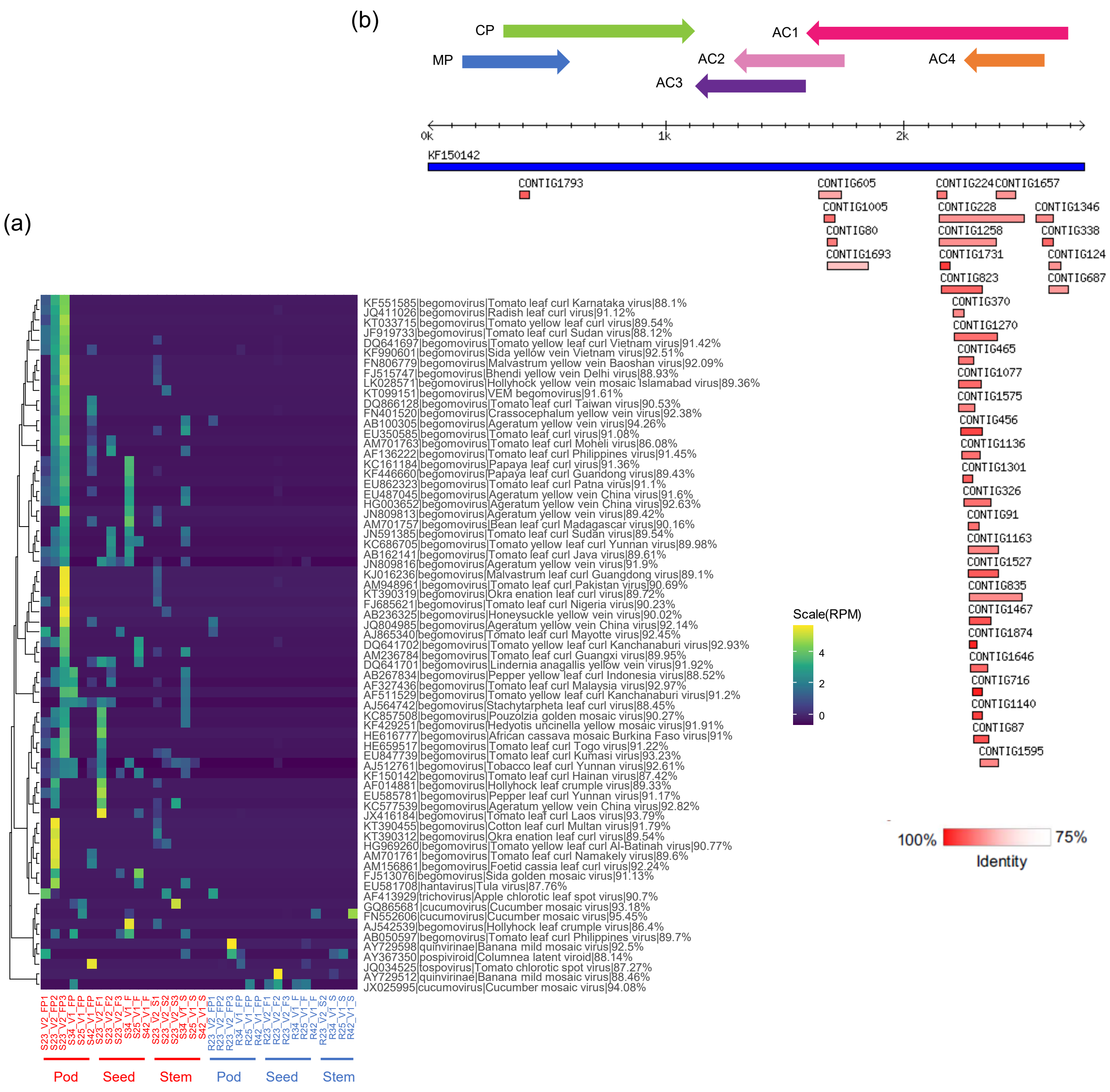

Figure S17 Viral contigs identified by VirusDetect using sRNA sequencing data. (a) Hierarchical clustered heatmap of differentially expressed viral contigs between healthy and SGS diseased samples across diverse soybean compartment niches. Differentially expressed viral contigs with |log2FC| > 2 (FC, fold change) and adjusted P-value < 0.01 were displayed in the heatmap. The name of each contig was labeled using the virus most resemblance to it identified by BLASTN. Red and blue labels represent diseased and healthy samples, respectively. The stem, pod, and seed samples of ZD23 was subjected to sRNA sequencing for virus identification. Originally, three repeats of sRNA for each sample group were prepared, but only one stem sRNA sample of healthy ZD23 (R23_V2_S2) was sucessfully extracted. As a complement, the stem, pod, and seed samples of ZD25, ZD34 and ZD42 were also subjected to sRNA sequencing (one repeat for each sample group). (b) Alignments of identified virus contigs to the reference virus genomes, using Tomato leaf curl Hainan virus isolate FQ12 (KF150142) as a example. The top arrows shows the position of the linearized genome organization (the open reading frames (ORFs) organization) in the alignment. Blue tracks represent reference virus genomes, and red tracks represent assembled virus contigs.
